# Metal-free selective mono-halodecarboxylation of heteroarenes under mild conditions

**DOI:** 10.1098/rsos.180333

**Published:** 2018-06-20

**Authors:** Scott H. Henderson, Ryan A. West, Simon E. Ward, Mark A. Honey

**Affiliations:** 1 Sussex Drug Discovery Centre, University of Sussex, Brighton BN1 9RH, UK; 2 Medicines Discovery Institute, Cardiff University, Park Place CF10 3AT, UK; 3 School of Pharmacy, Faculty of Science and Engineering, University of Wolverhampton, Wolverhampton WV1 1LY, UK

**Keywords:** halodecarboxylation, selective halogenation, mild conditions

## Abstract

The halodecarboxylation of heteroarene carboxylic acids by treatment with *N*-bromosuccinimide or *N*-chlorosuccinimide was performed. This procedure provides a convenient route to synthetically useful mono-halogenated heteroarene intermediates such as halo-indoles, -aza-indoles, -indazoles and -aza-indazoles. The mild conditions employed and simple protocol provides an advantage over traditional halodecarboxylation procedures that require expensive and toxic metal catalysts, basic conditions, time-consuming intermediate isolation and elevated reaction temperatures.

## 1. Introduction

**Figure 1.**
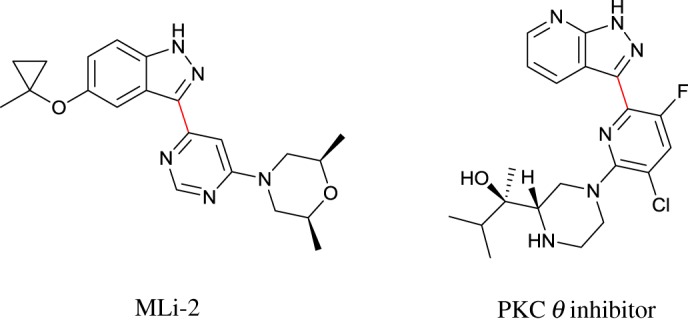
Chemical bonds in red can be constructed through cross-coupling with haloheteroarene precursor.

Haloheteroarenes serve as versatile building blocks in organic chemistry, most notably as coupling partners in transition metal-catalysed cross-coupling reactions for the formation of C–C and C–N bonds. Owing to their utility, haloheteroarenes have been used in the construction of molecules of biological interest including natural products [1–3] and small molecules in drug discovery programmes such as Merck's recent LRRK2 inhibitor, MLi-2 [4] and Vertex's PKC *θ* inhibitor (figure 1) [5].

**Figure 2.**
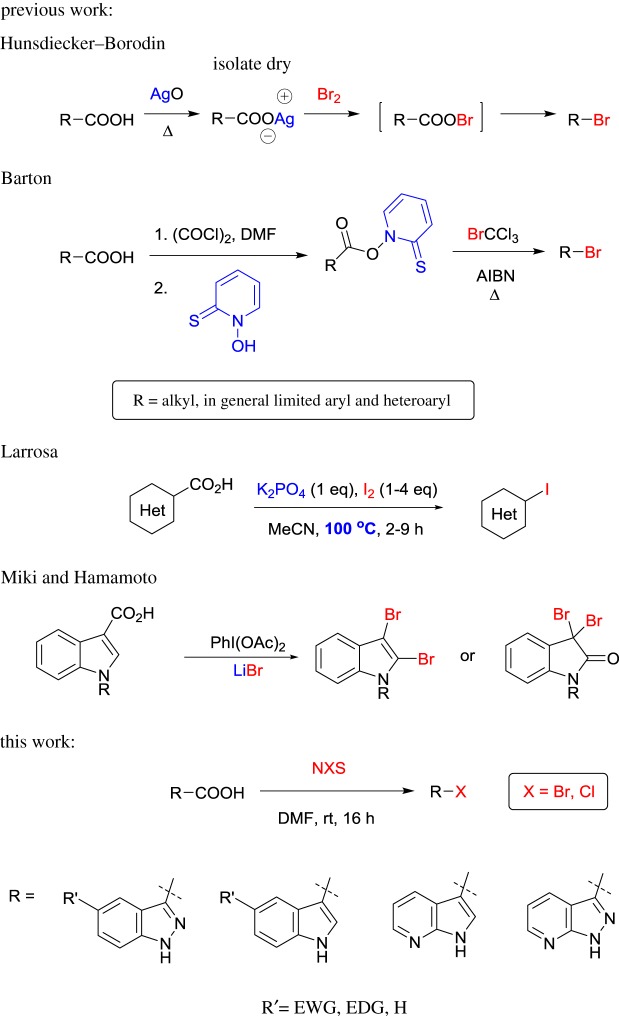
Strategies for halodecarboxylation [8,9].

Few methods are documented that describe the selective monohalogenation of heteroaromatic scaffolds [6–8]. A traditional approach to the regiospecific installation of a halogen atom on a substrate is the thermal halodecarboxylation of a silver(I) carboxylate salt in the presence of a halide source known as the Hunsdiecker–Borodin reaction (HBR) (figure 2) [10–12]. Substrates for the HBR are often aliphatic carboxylates with limited aromatic and heteroaromatic caboxylates being employed successfully [9]. The preparation of bromopyridines under basic aqueous conditions at elevated temperatures offers a rare example of the HBR being successfully applied to heterocyclic acid substrates [13,14]. The major shortcoming of the HBR is the requirement to isolate the dry silver carboxylate salt in sufficient purity to undergo decarboxylation and afford the corresponding halide in good yield [15]. Accordingly, modifications of the HBR such as the Cristol–Firth modification (CFM) and Kochi modification (KM) avoid the need to isolate the silver salt by employing more stable metal salts such as red mercury [16] and lead acetate [17] that undergo halodecarboxylation on heating *in situ*. The CFM and KM suffer from the same major limitation that they both employ toxic metal reagents to initiate the decarboxylation. The Barton modification avoids the use of toxic and expensive metals yet requires the preparation of the respective thiohydroxamate ester (Barton ester). Halodecarboxylation of the Barton ester results in the formation of both the desired halide and the undesired 2-(alkylthio)-pyridine side-product, limiting the atom efficiency of the reaction (figure 2) [18]. Recent advances in the field include the metal-free decarboxylative halogenation of unsaturated carboxylic acids with *N*-halosuccinimides in the presence of tetrabutylammonium trifluoroacetate [19], LiOAc [20] or photolysis with bromine and PhI(OAc)_2_ [21,22]. Hypervalent iodine has also been used by Hamamoto & Miki [23] for the synthesis of polybromoindoles. However, this methodology is seemingly unable to produce mono-halogenated products and requires the use of alkali metal salts as the halide source. Since preparing this manuscript, a recent report by Larrosa [24] has described the decarboxylative iodination of benzoic and heterobenzoic acids through the use of iodine and potassium phosphate. Although this protocol is very diverse, it requires the use of stoichiometric amounts of base, elevated temperatures (100°C) and currently suffers from over bromination (when bromine is used in place of iodine) of substrates owing to the increased electrophilicity of bromine in comparison to that of iodine.

With the above in mind, current methods of halodecarboxylation suffer from the use of highly toxic and expensive metals employed in stoichiometric amounts, requires the use of corrosive elemental halide sources or suffer over halogenation. It is, therefore, desirable to establish a more reliable and economical method for mono-halodecarboxylation of heteroarene carboxylic acids to furnish synthetically useful haloheteroarenes. Presently, we describe a simple base-free method for the regiospecific mono-halodecarboxylation of substituted and unsubstituted heteroarene carboxylic acids under mild and metal-free conditions at room temperature with *N*-bromosuccinimide (NBS) and *N*-chlorosuccinimide (NCS).

## 2. Results and discussion

Our interest in the synthesis of haloheteroarene precursors peaked when exploring indazoles as a potential scaffold for an in-house kinase inhibitor project. Our studies began somewhat fortuitously with the bromodecarboxylation of indazole **1** with NBS. The reaction proceeded with two equivalents of NBS in *N,N*-dimethylformamide (DMF) at room temperature affording product **2** in 35% yield (table 1, entry 1) after just 1 h. Inspection of the reaction mixture by liquid chromatography-mass spectrometry (LCMS) analysis appeared to show that a dibrominated product had also formed as a major impurity. Gratifyingly, by decreasing the equivalents of NBS it was observed that no measurable amount of dibrominated product was formed. Despite the reaction being facile it was found that by extending the reaction time from 1 to 16 h, the reaction reliably went to completion. Under the optimized conditions product **2** was furnished in 77% isolated yield (table 1, entry 2).

**Table 1.** Screening of optimal conditions. Reaction conditions: 1 (0.2 mmol), NBS, solvent (1.5 ml) at room temperature (rt). 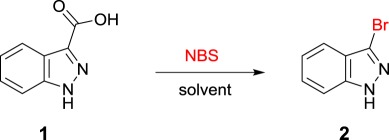

entry	solvent	NBS (equiv.)	reaction time (h)	isolated yield (%)	% conversion by LCMS
1	DMF	2	1	35	35
2	DMF	1	16	77	86
3	DCM	1	16	—	trace product
4	EtOAc	1	16	—	50
5	MeCN	1	16	—	75

Reactions involving NBS can be exothermic and can pose a risk when undertaken on a large scale [25], therefore, we sought to employ a solvent that was predicted to be safe to use on a large scale and in addition would furnish the desired product in high yield. A small solvent screen was undertaken with solvents that were reported to cause negligible exotherms when used with NBS (table 1, entries 3–5) [25]. Unfortunately, none of these solvents facilitated the halodecarboxylation reaction in as high yield as observed when the reaction was carried out in DMF (table 1).

**Figure 3.**
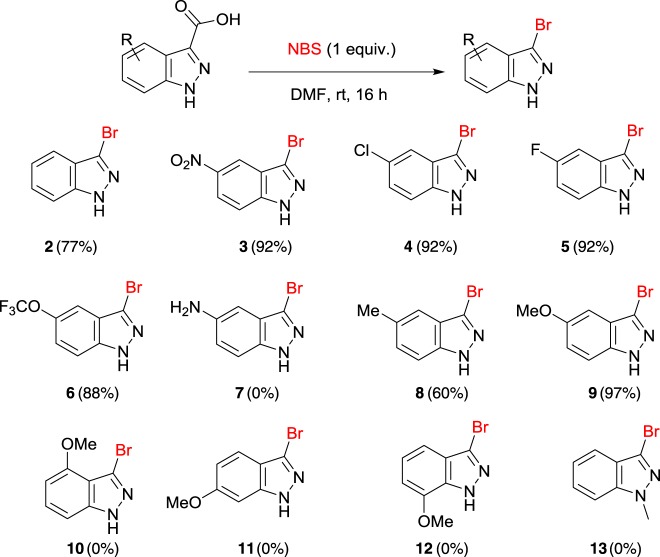
Halodecarboxylation of indazoles. Reaction conditions: heteroarene carboxylic acid (0.2 mmol), NBS (0.2 mmol), solvent (1.5 ml) at room temperature (rt).

With the halodecarboxylation procedure in hand, the indazole carboxylic acid scope was investigated (figure 3). A variety of indazole carboxylic acids were subjected to the reaction with NBS in DMF (products **2**–**13**). Examples of indazole acids bearing electron withdrawing (products **3**–**5**) and electron donating substituents (products **8** and **9**) underwent decarboxylative bromination in excellent yields under the reaction conditions. However, when the electron donating methoxy group was moved to the 4, 6 and 7 positions on the indazole (**10**, **11** and **12**), no product could be detected. The use of *N*-methyindazole to afford compound **13** appeared to be unsuccessful, with no halodecarboxylated product observed by LCMS—it appears as though the presence of the free indazole N–H is a requirement for decarboxylation when using the current set of reaction conditions. Halodecarboxylation to afford the 5-amino indazole analogue (product **7**) also proved unsuccessful. Initial mechanistic investigations reveal that the reaction possibly proceeds through a non-radical pathway, as the reaction continued to proceed when performed in the absence of light. However, detailed mechanistic investigations are continuing within our laboratory, and so a clearer insight into the lack of reactivity of some substrates will be detailed in due course.

We now turned our attention to chlorodecarboxylation and iododecarboxylation reactions using the optimized conditions but employing NCS and *N*-iodosuccinimide (NIS) as halide sources (table 2, entries 2 and 3). Although the chlorodecarboxylation product (table 2, entry 2) was obtained in lower yield than the bromodecarboxylation product (table 2, entry 1) using similar conditions, the current procedure represents one of the few ways to synthesize chloroheteroarenes under mild conditions. Unfortunately, the iododecarboxylation was not facilitated under the current reaction conditions.

**Table 2.** Screening of halodecarboxylation conditions. Reaction conditions: 1 (0.2 mmol), *N*-halosuccinimide, DMF (1.5 ml) at room temperature (rt). 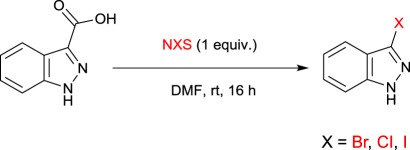

entry	NXS	isolated yield (%)	% conversion by LCMS
1	NBS	77	86
2	NCS	39	35
3	NIS	—	0

**Figure 4.**
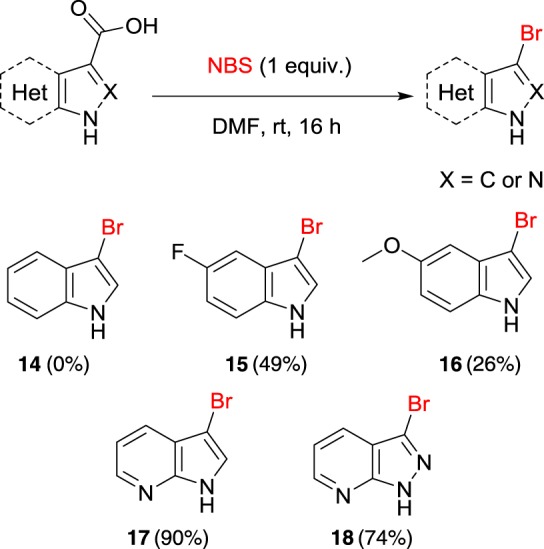
Halodecarboxylation of heteroarene carboxylic acids. Reaction conditions: heteroarene carboxylic acid (0.2 mmol), NBS (0.2 mmol), solvent (1.5 ml) at room temperature (rt).

To demonstrate the versatility of the halodecarboxylation procedure for heteroarenes, further reactions with NBS were investigated (figure 4). Although halo-indoles (products **14**–**16**) have been reported in the literature to be problematic to isolate [26,27], under the current procedure we have been able to furnish **15** and **16** in reasonable yields.

Aza-indole and aza-indazole acids underwent halodecarboxylation smoothly to furnish the corresponding haloheteroarenes **17** and **18** in good yield, implying that more electron deficient systems undergo halodecarboxylation efficiently under the current conditions. Attempts to further expand this methodology to simple benzoic acids have currently proved unsuccessful, although this remains an active area of research within our group.

## 3. Conclusion

In summary, a mild and efficient protocol for the mono-halodecarboxylation of heteroarene carboxylic acids has been developed. It is noteworthy that this halodecarboxylation is metal-free and displays significant advantages over traditional methods of halodecarboxylation that commonly require harsh reaction conditions, toxic reagents or suffer over bromination. The proposed method extends the substrate scope of traditional halodecarboxylations to heteroarene substrates. This methodology provides a novel and direct route to haloheteroarene building blocks that can be used in transition metal-catalysed cross-coupling reactions to construct molecules of scientific importance. Investigations into the site-selective halodecarboxylation of other heteroarene acid precursors are currently underway in our laboratory.

## Supplementary Material

Spectroscopic data for synthesised compounds

## Data accessibility

Experimental data associated with this article can be found as part of the electronic supplementary material.

## References

[1] Joules JA, Mills K 2010 Heterocyclic chemistry, fifth edition Chichester, UK: John Wiley and Sons.

[2] Gribble GW 2004 Natural organohalogens: a new frontier for medicinal agents? J. Chem. Educ. 81, 1441 (doi:10.1021/ed081p1441)

[3] Carbain B, Hitchcock PB, Streicher H 2010 New aspects of the Hunsdiecker–Barton halodecarboxylation: syntheses of phospha-shikimic acid and derivatives. Tetrahedron Lett. 51, 2717–2719. (doi:10.1016/j.tetlet.2010.03.044)

[4] Scott JD et al. 2017 Discovery of a 3-(4-pyrimidinyl) indazole (MLi-2), an orally available and selective leucine-rich repeat kinase 2 (LRRK2) inhibitor that reduces brain kinase activity. J. Med. Chem. 60, 2983–2992. (doi:10.1021/acs.jmedchem.7b00045) 28245354 10.1021/acs.jmedchem.7b00045

[5] Jimenez J-M et al. 2013 Design and optimization of selective protein kinase C *θ* (PKC*θ*) inhibitors for the treatment of autoimmune diseases. J. Med. Chem. 56, 1799–1810. (doi:10.1021/jm301465a) 23398373 10.1021/jm301465a

[6] Ganguly NC, De P, Dutta S 2005 Mild regioselective monobromination of activated aromatics and hetero­aromatics with *N*-bromosuccinimide in tetrabutylammonium bromide. Synthesis 7, 1103–1108. (doi:10.1055/s-2005-861866)

[7] Grimmett R 1994 Halogenation of heterocycles: III. Heterocycles fused to other aromatic or heteroaromatic rings, advances in heterocyclic chemistry. In Advances in heterocyclic chemistry (ed. Katritzky AR), vol. 59, pp. 245–369. New York, NY: Elsevier.

[8] Eisch JJ 1967 Halogenation of heterocyclic compounds, advances in heterocyclic chemistry. In Advances in heterocyclic chemistry (eds Katritzky AR, Boulton AJ), vol. 7, p. 1 New York, NY: Elsevier.

[9] Johnson RG, Ingham RK 1956 The degradation of carboxylic acid salts by means of halogen: the Hunsdiecker reaction. Chem. Rev. 56, 219–269. (doi:10.1021/cr50008a002)

[10] Borodin B 1861 Ueber Bromvaleriansaure und brombuttersaure. Annalen der Chemie und Pharmacie 119, 121–123. (doi:10.1002/jlac.18611190113)

[11] Hunsdiecker HC 1942 Über den Abbau der Salze aliphatischer Säuren durch Brom. Chem. Ber. 75, 291 (doi:10.1002/cber.19420750309)

[12] Kürt L, Czakó B 2005 Strategic applications of named reactions in organic synthesis: background and detailed mechanisms. Burlington, MA: Elsevier Academic Press.

[13] Kuffner F, Russo C 1954 Versuche über den Verlauf des Silbersalzabbaues mittels Brom bei den Pyridin-monocarbonsäuren. Monatshefte Chem. 85, 1097–1103. (doi:10.1007/BF00899858)

[14] Mariella RP, Belcher EP 1952 α-Oxygenated pyridines. III. The reaction of N-bromosuccinimide with some pyridine derivatives. J. Am. Chem. Soc. 74, 1916–1919. (doi:10.1021/ja01128a013)

[15] Laue T, Plagens A 2005 Named organic reactions, 2nd Edn Chichester, UK: John Wiley and Sons, Ltd.

[16] Cristol S, Firth W 1961 Communications. a convenient synthesis of alkyl halides from carboxylic acids. J. Org Chem. 26, 280 (doi:10.1021/jo01060a628)

[17] Kochi JK 1965 Formation of alkyl halides from acids by decarboxylation with lead(IV) acetate and halide salts. J. Org. Chem. 30, 3265–3271. (doi:10.1021/jo01021a002)

[18] Barton DHR, Lacher B, Zard SZ 1985 Radical decarboxylative bromination of aromatic acids. Tetrahedron Lett. 26, 5939–5942. (doi:10.1016/S0040-4039(00)98266-2)

[19] Roy S 2000 Catalytic Hunsdiecker reaction and one-pot catalytic Hunsdiecker–Heck strategy: synthesis of *α*,β-unsaturated aromatic halides, α-(dihalomethyl)benzenemethanols, 5-aryl-2,4-pentadienoic acids, dienoates and dienamides. Tetrahedron 56, 1369–1377. (doi:10.1016/S0040-4020(99)01035-2)

[20] Chowdhury S, Roy S 1997 The first example of a catalytic hunsdiecker reaction: synthesis of β-halostyrenes. J. Org. Chem. 62, 199–200. (doi:10.1021/jo951991f) 11671382 10.1021/jo951991f

[21] Concepcion JI, Francisco CG, Freire R, Hernandez R, Salazar JA, Suarez E 1986 Iodosobenzene diacetate, an efficient reagent for the oxidative decarboxylation of carboxylic acids. J. Org. Chem. 51, 402–404. (doi:10.1021/jo00353a026)

[22] Moriarty RM, Khosrowshahi JS, Dalecki TM 1987 Hypervalent iodine iodinative decarboxylation of cubyl and homocubyl carboxylic acids. J.C.S. Chem. Comm. 9, 675–676. (doi:10.1039/C39870000675)

[23] Hamamoto H, Umemoto H, Umemoto M, Ohta C, Fujita E, Nakamura A, Maegawa T, Miki Y 2015 Decarboxylative halogenation of indolecarboxylic acids using hypervalent iodine(III) reagent and its application to the synthesis of polybromoindoles. Heterocycles 91, 561–572. (doi:10.3987/COM-14-13162)

[24] Perry GJP, Quibell JM, Panigrahi A, Larrosa I 2017 Transition-metal-free decarboxylative iodination: new routes for decarboxylative oxidative cross-couplings. J. Am. Chem. Soc, 139, 115 27–115 36. (doi:10.1021/jacs.7b05155) 28735532 10.1021/jacs.7b05155 PMC5662929

[25] Shimizu S, Imamura Y, Ueki T 2014 Incompatibilities between *N*-bromosuccinimide and solvents. Org. Process Res. Dev. 18, 354–358. (doi:10.1021/op400360k)

[26] Piers K, Meimaroglou C, Jardine RV, Brown RK 1963 The preparation of 3-bromoindole. Can. J. Chem. 41, 2399–2401. (doi:10.1139/v63-353)

[27] Weissgerber R 1913 Zur Kenntnis des Indols. Ber. Dtsch Chem. Ges. 46, 651 (doi:10.1002/cber.19130460188)

