# Supplementary material for: Metal-free selective mono-halodecarboxylation of heteroarenes under mild conditions
Source: R Soc Open Sci. 2018 Jun 20;5(6):180333. doi: 10.1098/rsos.180333 (PMC6030312; doi:10.1098/rsos.180333)

## General information

All commercial reagents were purchased from Sigma-Aldrich, Alfa Aesar, Apollo Scientific, Fluorochem or Tokyo Chemical Industry and of the highest available purity. *N*-bromosuccinimide (NBS) and *N*-chlorosuccinimide (NCS) were recrystallized from water. Unless otherwise stated, chemicals were used as supplied without further purification. Anhydrous solvents were purchased from Acros (AcroSeal™) or Sigma-Aldrich (SureSeal™) and were stored under nitrogen. Anhydrous solvents and reagents were used as purchased. Thin layer chromatography (TLC) was carried out using glass plates pre-coated with Merck silica gel 60 F254. Melting points were determined using an OptiMelt apparatus and are uncorrected. Proton nuclear magnetic resonance spectra were recorded at 500 MHz on a Varian VNMRS 500 MHz spectrometer or at 500 MHz on a Bruker Biospin GmbH 500 MHz spectrometer or at 600 MHz on a Varian VNMRS 600 MHz spectrometer (at 30 °C), using residual isotopic solvent ($CHCl_3$, δH = 7.27 ppm, DMSO δH = 2.50 ppm, MeOH δH = 3.31 ppm) as an internal reference. Chemical shifts are quoted in parts per million (ppm). Coupling constants (*J*) are recorded in Hertz (Hz). Carbon nuclear magnetic resonance spectra were recorded at 125 MHz on a Varian 500 MHz spectrometer or 151 MHz on a Varian 600 MHz spectrometer and are proton decoupled, using residual isotopic solvent ($CHCl_3$, δC = 77.00 ppm, DMSO δC = 39.52 ppm, MeOH δC = 49.00 ppm) as an internal reference. Proton and carbon spectra assignments are supported by DEPT editing. Chemical shifts (δC) are quoted in ppm. High resolution mass spectrometry (HRMS) data (ESI) was recorded on Bruker Daltonics, Apex III, ESI source: Apollo ESI with methanol as spray solvent. Only molecular ions, fractions from molecular ions and other major peaks are reported as mass/charge (m/z) ratios. UPLCMS data was recorded on a Waters Acquity SDS ZQMS. Samples were eluted through a Kinetex 2.6 µm C18 50 mm x 2.1 mm column, using water and acetonitrile acidified by 0.1% formic acid at 1 ml/min and detected at 254 nm. The gradient employed was a 1.5 min method 2–95% MeCN over a 1.45 min gradient, then re-equilibrated to 2% MeCN over 0.5 min. LCMS data was recorded on a Waters 2695 HPLC using a Waters 2487 UV detector and a Thermo LCQ ESI-MS. Samples were eluted through a Phenomenex Lunar 3 µm C18 50 mm × 4.6 mm column, using water and acetonitrile acidified by 0.1% formic acid at 1 ml/min and detected at 254 nm. The gradient employed was a 7 min method 30–90% MeCN over a 5 min gradient, held at 90% MeCN for 1 min, then re-equilibrated to 30% MeCN over 1 min. All experiments were carried out in sealed microwave vials.

## Synthesis of 3-bromo-1*H*-indazole. General procedure.

To a microwave vial containing a stirred suspension of 1*H*-indazole-3-carboxylic acid (0.280 mmol) in DMF (1.5 mL) was added *N*-bromosuccinimide (0.280 mmol). The microwave vial was sealed and the reaction mixture was stirred at room temperature for 16 h. Purification of the reaction mixture by reverse phase column chromatography afforded 3-bromo-1*H*-indazole.

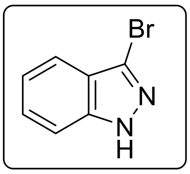


**3-bromo-1*H*-indazole (2):** colourless solid; mp = 142-143 °C (lit., 141-142 °C)*; yield: 47 mg (77%); $^1$H NMR (500 MHz, DMSO-$d_6$) δ 13.43 (s, 1H), 7.57 (d, *J* = 8.4 Hz, 2H), 7.45 (t, *J* = 7.5 Hz, 1H), 7.22 (t, *J* = 7.5 Hz, 1H); $^{13}$C NMR (151 MHz, DMSO-$d_6$) δ 141.0, 127.5, 122.1, 121.5, 120.4, 119.2, 110.9; HRMS (ESI) m/z calculated for $C_7H_4BrN_2$ $[M-H]^-$ 194.9563, found 194.9558.

* v. Auwers, L. *Chemische Berichte*. 1922. 55, 1141.

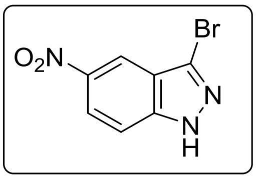

**3-bromo-5-nitro-1*H*-indazole (3):** yellow solid; mp = 222-224 °C (lit., 223-225 °C)*; yield: 53.7 mg (92%); $^{1}$H NMR (500 MHz, DMSO-$d_6$) δ 8.53 – 8.45 (m, 1H), 8.26 (dd, *J* = 9.2, 2.4 Hz, 1H), 7.79 (dd, *J* = 9.2 Hz, 0.5 Hz, 1H); $^{13}$C NMR (151 MHz, DMSO-$d_6$) δ 143.0, 142.2, 123.6, 122.2, 121.5, 117.0, 112.2; HRMS (ESI) m/z calculated for $C_7H_3BrN_3O_2$ [M-H]$^-$ 239.9414, found 239.9405.

* Benchidmi, M *et al*. *Journal of Heterocyclic Chemistry*. 1979. 16, 1599.

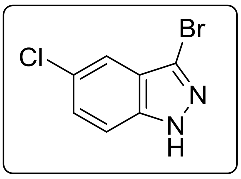

**3-bromo-5-chloro-1*H*-indazole (4):** colourless solid; mp = 211-213 °C (lit., 212 °C)*; yield: 54.2 mg (92%); $^{1}$H NMR (500 MHz, DMSO-$d_6$) δ 13.64 (s, 1H), 7.66 – 7.59 (m, 2H), 7.45 (dd, *J* = 8.9, 2.0 Hz, 1H); $^{13}$C NMR (151 MHz, DMSO-$d_6$) δ 139.6, 128.0, 126.0, 123.0, 119.7, 118.3, 112.8; HRMS (ESI) m/z calculated for $C_7H_3BrClN_2$ [M-H]$^-$ 228.9174, found 228.9166.

* Lohou, E; Collot, V; Stiebing, S; Rault, S. *Synthesis*. 2011, 16, 2651.

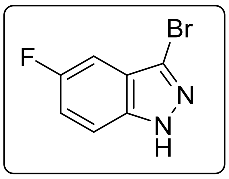

**3-bromo-5-fluoro-1*H*-indazole (5):** colourless solid; mp = 189-191 °C; yield: 55 mg (92%); $^{1}$H NMR (500 MHz, DMSO-$d_6$) δ 13.57 (s, 1H), 7.67 – 7.58 (m, 1H), 7.39 – 7.32 (m, 2H); $^{13}$C NMR (151 MHz, DMSO-$d_6$) δ 157.5 (d, *J* = 237.4 Hz), 138.1, 122.04 (d, *J* = 11.0 Hz), 119.9 (d, *J* = 5.8 Hz), 117.1 (d, *J* = 27.7 Hz), 112.7 (d, *J* = 9.7 Hz), 103.2 (d, *J* = 24.6 Hz); HRMS (ESI) m/z calculated for $C_7H_3BrFN_2$ [M-H]$^-$ 212.9469, found 212.9464.

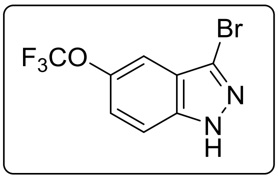

**3-bromo-5-(trifluoromethoxy)-1*H*-indazole (6):** colourless solid; mp = 149-151 °C; yield: 50 mg (88%); $^{1}$H NMR (500 MHz, DMSO-$d_6$) δ 7.71 (d, *J* = 8.9 Hz, 1H), 7.57 (s, 1H), 7.46 (d, *J* = 8.9 Hz, 1H); $^{13}$C NMR (151 MHz, DMSO-$d_6$) δ 143.0, 139.6, 122.1, 121.2, 120.6, 119.5, 113.0, 111.4; HRMS (ESI) m/z calculated for $C_8H_3BrF_3N_2O$ [M-H]$^-$ 278.9386, found 278.9378.

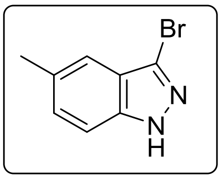


**3-bromo-5-methyl-1*H*-indazole (8):** colourless solid; mp = 157-159 °C (lit., 158-159 °C)*; yield: 38 mg (60%); $^{1}$H NMR (600 MHz, Chloroform-*d*) δ 10.56 (s, 1H), 7.47 – 7.39 (m, 2H), 7.30 (dd, *J* = 8.7, 1.4 Hz, 1H), 2.50 (s, 3H); $^{13}$C NMR (151 MHz, $CDCl_3$) δ 139.8, 131.5, 130.2, 123.3, 122.3, 119.1, 109.8, 21.3; LRMS (ESI) m/z 211.1 $(M+H)^+$.

* v. Auwers, L. *Chemische Berichte*. 1922, 55, 1141.

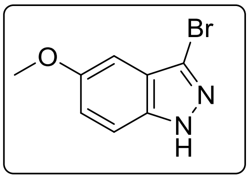


**3-bromo-5-methoxy-1*H*-indazole (9):** colourless solid; mp = 178-180 °C (lit., 178 °C)*; yield: 57 mg (97%); $^{1}$H NMR (500 MHz, DMSO-$d_6$) δ 13.29 (s, 1H), 7.48 (d, *J* = 9.0 Hz, 1H), 7.08 (dd, *J* = 9.0, 2.5 Hz, 1H), 6.95 – 6.82 (m, 1H), 3.81 (s, 3H); $^{13}$C NMR (151 MHz, DMSO-$d_6$) δ 154.8, 136.8, 122.3, 119.9, 119.4, 112.1, 97.9, 55.5; HRMS (ESI) m/z calculated for $C_8H_6BrN_2O$ $[M-H]^-$ 224.9669, found 224.9962.

* Lohou, E; Collot, V; Stiebing, S; Rault, S. *Synthesis*. 2011, 16, 2651.

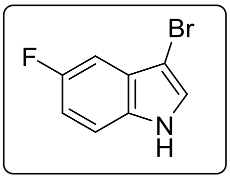


**3-bromo-5-fluoro-1*H*-indole (12):** pale brown solid; yield: 32 mg (49%); $^{1}$H NMR (500MHz, DMSO-$d_6$) δH 11.32 (s, 1H), 7.48 (d, *J* = 2.6 Hz, 1H), 7.33 (d, *J* = 8.7 Hz, 1H), 6.91-6.74 (m, 2H), 3.79 (s, 3H).

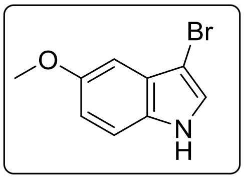


**3-bromo-5-methoxy-1*H*-indole (13):** pale brown solid; mp = 79-81 °C; yield: 18 mg (26%); $^{1}$H NMR (500MHz, DMSO-$d_6$) δH 11.59 (s, 1H), 7.64 (d, *J* = 2.7 Hz, 1H), 7.45 (dd, *J* = 8.9, 4.4 Hz, 1H), 7.21 (dd, *J* = 9.3, 2.5 Hz, 1H), 7.04 (td, *J* = 9.3, 2.5 Hz, 1H); LRMS (ESI) m/z 226.3 $(M+H)^+$.

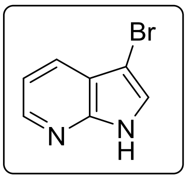


**3-bromo-1*H*-pyrrolo[2,3-*b*]pyridine (14):** pale brown solid; mp = 187-189 °C (lit., 188-188.5 °C)*; yield: 55 mg (90%); $^{1}$H NMR (500MHz, DMSO-$d_6$) δ 12.07 (s, 1H), 8.29 (d, *J* = 4.6 Hz, 1H), 7.84 (d, *J* = 7.9 Hz, 1H), 7.71 (s, 1H), 7.18 (dd, *J* = 7.9, 4.6 Hz, 1H); $^{13}$C NMR (151 MHz, DMSO-$d_6$) δ 147.2, 143.9,

126.5, 125.6, 118.7, 116.4, 87.2; HRMS (ESI) m/z calculated for $C_7H_6BrN_2$ $[M+H]^+$ 196.9709, found 196.9708.

* Robinson, M.M; Robinson, B.L. *Journal of the American Chemical Society*. 1956, 78, 1247.

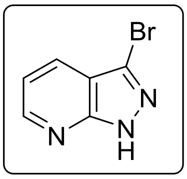


**3-bromo-1*H*-pyrazolo[3,4-*b*]pyridine (15):** colourless solid; mp = 165-167 °C (lit., 165-166 °C)*; yield: 45 mg (74%); $^1$H NMR (500MHz, DMSO-$d_6$) δH 8.61 (dd, *J* = 4.5, 1.9 Hz, 1H), 8.10 (dd, *J* = 8.1, 1.9 Hz, 1H), 7.30 (dd, *J* = 8.1, 4.5 Hz, 1H); $^{13}$C NMR (151 MHz, DMSO-$d_6$) δ 151.8, 150.4, 128.9, 119.4, 117.8, 114.4; LRMS (ESI) m/z 197.9 $(M+H)^+$.

* Kočevar, M; Stanovnik, B; Tišler, M. *Journal of Heterocyclic Chemistry*. 1978, 15, 1175.

To a microwave vial containing a stirred suspension of 1*H*-indazole-3-carboxylic acid (0.280 mmol) in DMF (1.5 mL) was added *N*-chlorosuccinimide (0.2800mmol). The microwave vial was sealed and the reaction mixture was stirred at room temperature for 16 h.  Purification of the reaction mixture by reverse phase column chromatography afforded 3-chloro-1*H*-indazole.

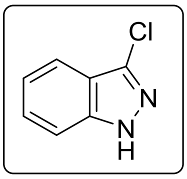


**3-chloro-1*H*-indazole:** colourless solid; mp = 147-148 °C (lit., 148 °C)*; yield: 20 mg (39%); $^1$H NMR (600MHz, DMSO-$d_6$) δ 13.32 (s, 1H), 7.66 (d, *J* = 8.2 Hz, 1H), 7.57 (d, *J* = 8.2 Hz, 1H), 7.48 – 7.43 (m, 1H), 7.26 – 7.20 (m, 1H); $^{13}$C NMR (151 MHz, DMSO-$d_6$) δ 141.0, 132.1, 127.5, 121.4, 119.4, 118.6, 111.0; HRMS (ESI) m/z calculated for $C_7H_4ClN_2$ $[M-H]^-$ 151.0068, found 151.0065.

* Fischer,E.; Seuffert, O. *Chemische Berichte*, 1901, 34, 798.

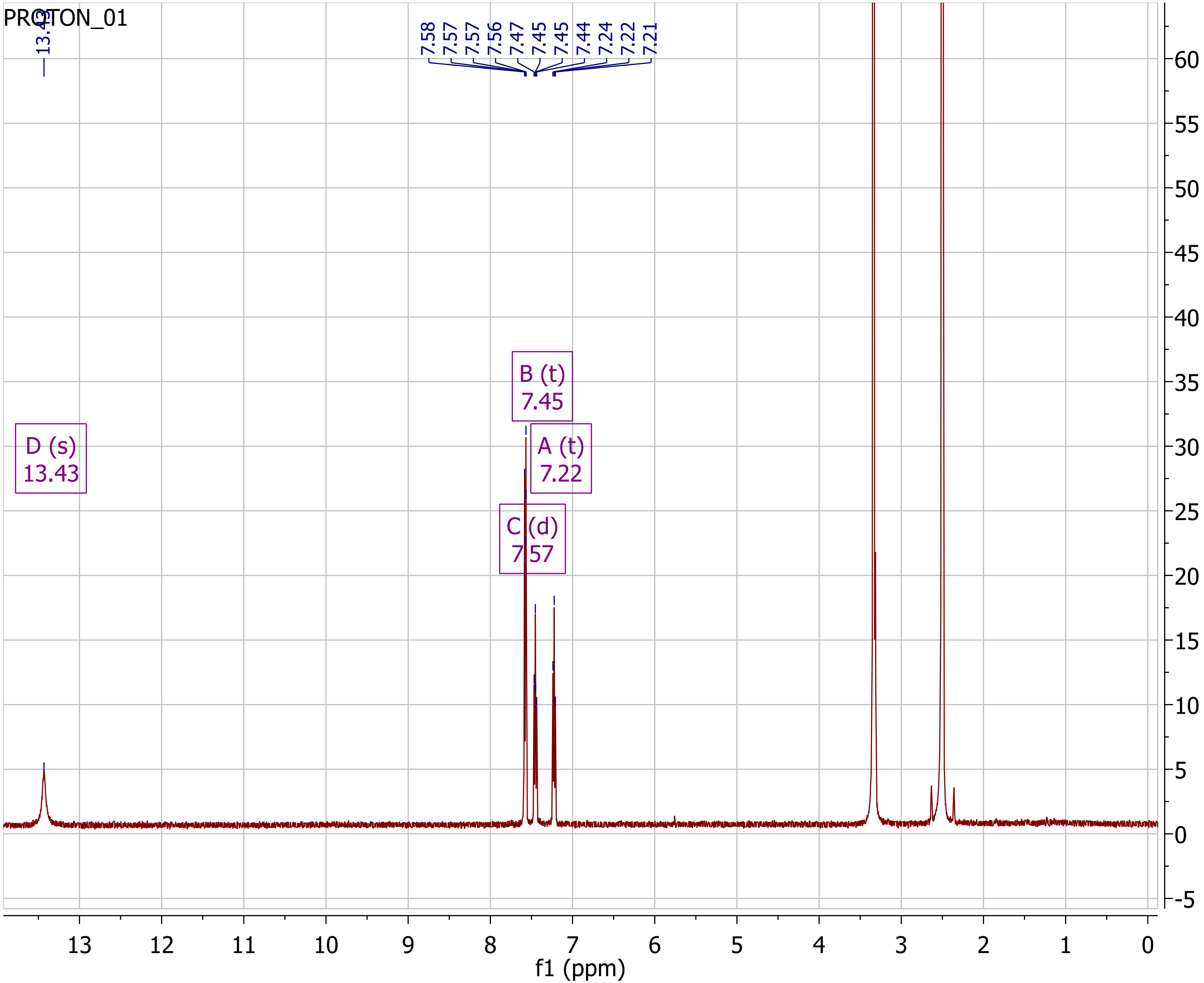


Sussex Drug Discovery Centre

| | Parameter | Value |
|---|---|---|
| 1 | Data File Name | Y:/ walkup/ sew/ 20171004/ N2118-108_01/ PROTON_01.fid/ fid |
| 2 | Title | PROTON_01 |
| 3 | Comment | |
| 4 | Origin | Varian |
| 5 | Owner | |
| 6 | Site | |
| 7 | Spectrometer | vnmrs |
| 8 | Author | |
| 9 | Solvent | dmso |
| 10 | Temperature | 30.0 |
| 11 | Pulse Sequence | s2pul |
| 12 | Experiment | 1D |
| 13 | Probe | P8898_walkup |
| 14 | Number of Scans | 8 |
| 15 | Receiver Gain | 44 |
| 16 | Relaxation Delay | 1.0000 |
| 17 | Pulse Width | 4.3000 |
| 18 | Presaturation Frequency | |
| 19 | Acquisition Time | 2.0447 |
| 20 | Acquisition Date | 2017-10-04T08:33:26 |
| 21 | Modification Date | 2017-10-04T08:33:55 |
| 22 | Class | |
| 23 | Spectrometer Frequency | 499.91 |
| 24 | Spectral Width | 8012.8 |
| 25 | Lowest Frequency | -996.5 |
| 26 | Nucleus | 1H |

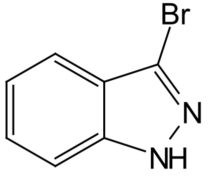


$^1$H NMR (500 MHz, DMSO-$d_6$) δ 13.43 (s, 1H), 7.57 (d, $J$ = 8.4 Hz, 2H), 7.45 (t, $J$ = 7.5 Hz, 1H), 7.22 (t, $J$ = 7.5 Hz, 1H).

Sussex Drug Discovery Centre

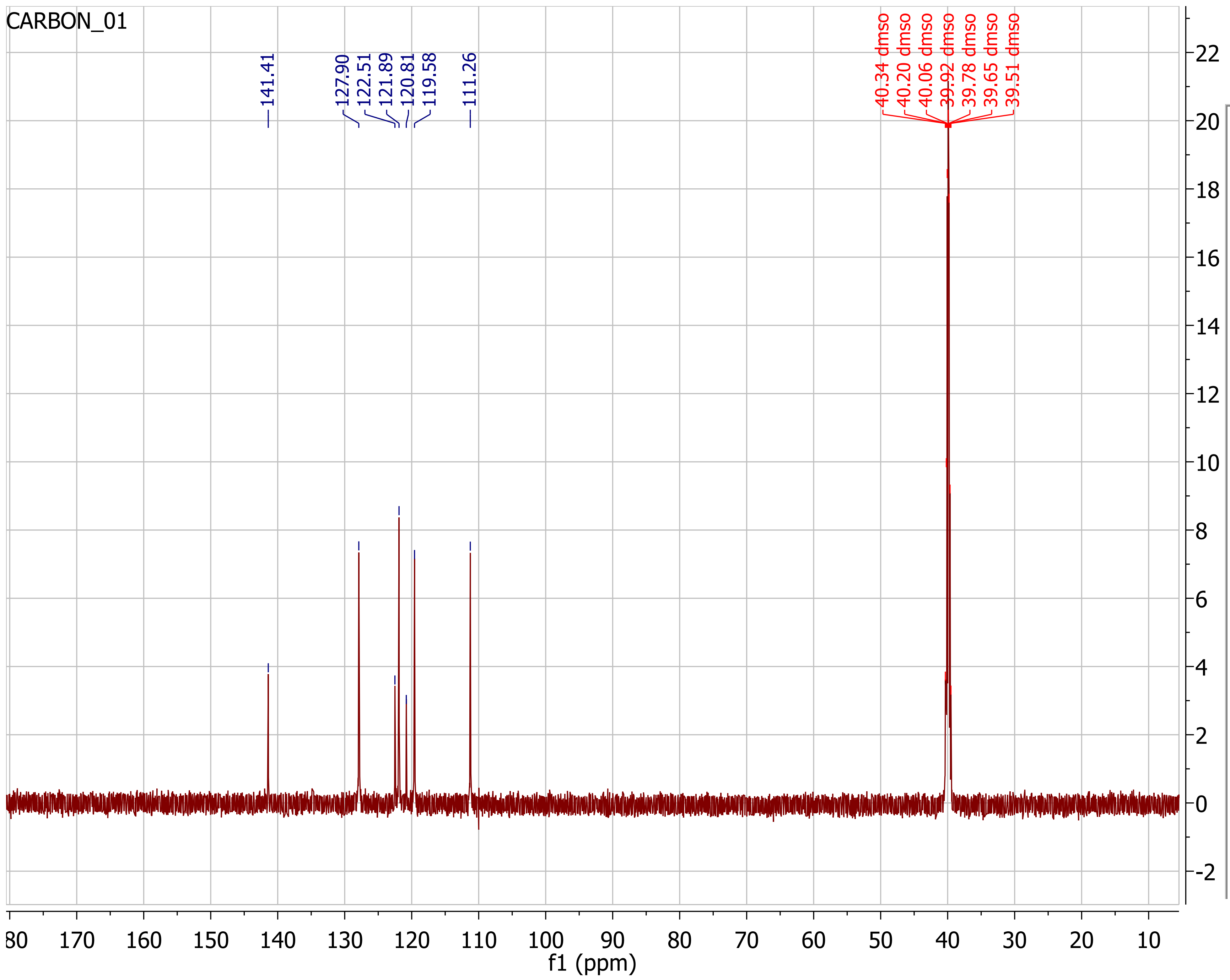


| | Parameter | Value |
|---|---|---|
| 1 | Data File Name | F:/ DB Project/ NMR/ Conc C NMR/ N2118-108_02/ CARBON_01.fid/ fid |
| 2 | Title | CARBON_01 |
| 3 | Comment | |
| 4 | Origin | Varian |
| 5 | Owner | |
| 6 | Site | |
| 7 | Spectrometer | vnmrs |
| 8 | Author | |
| 9 | Solvent | dmso |
| 10 | Temperature | 25.0 |
| 11 | Pulse Sequence | s2pul |
| 12 | Experiment | 1D |
| 13 | Probe | P8891 |
| 14 | Number of Scans | 256 |
| 15 | Receiver Gain | 30 |
| 16 | Relaxation Delay | 1.0000 |
| 17 | Pulse Width | 4.1500 |
| 18 | Presaturation Frequency | |
| 19 | Acquisition Time | 0.8651 |
| 20 | Acquisition Date | 2018-01-24T11:27:17 |
| 21 | Modification Date | 2018-01-24T11:35:20 |
| 22 | Class | |
| 23 | Spectrometer Frequency | 150.81 |
| 24 | Spectral Width | 37878.8 |
| 25 | Lowest Frequency | -2413.2 |
| 26 | Nucleus | 13C |

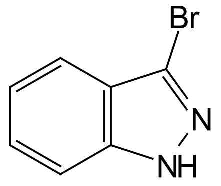

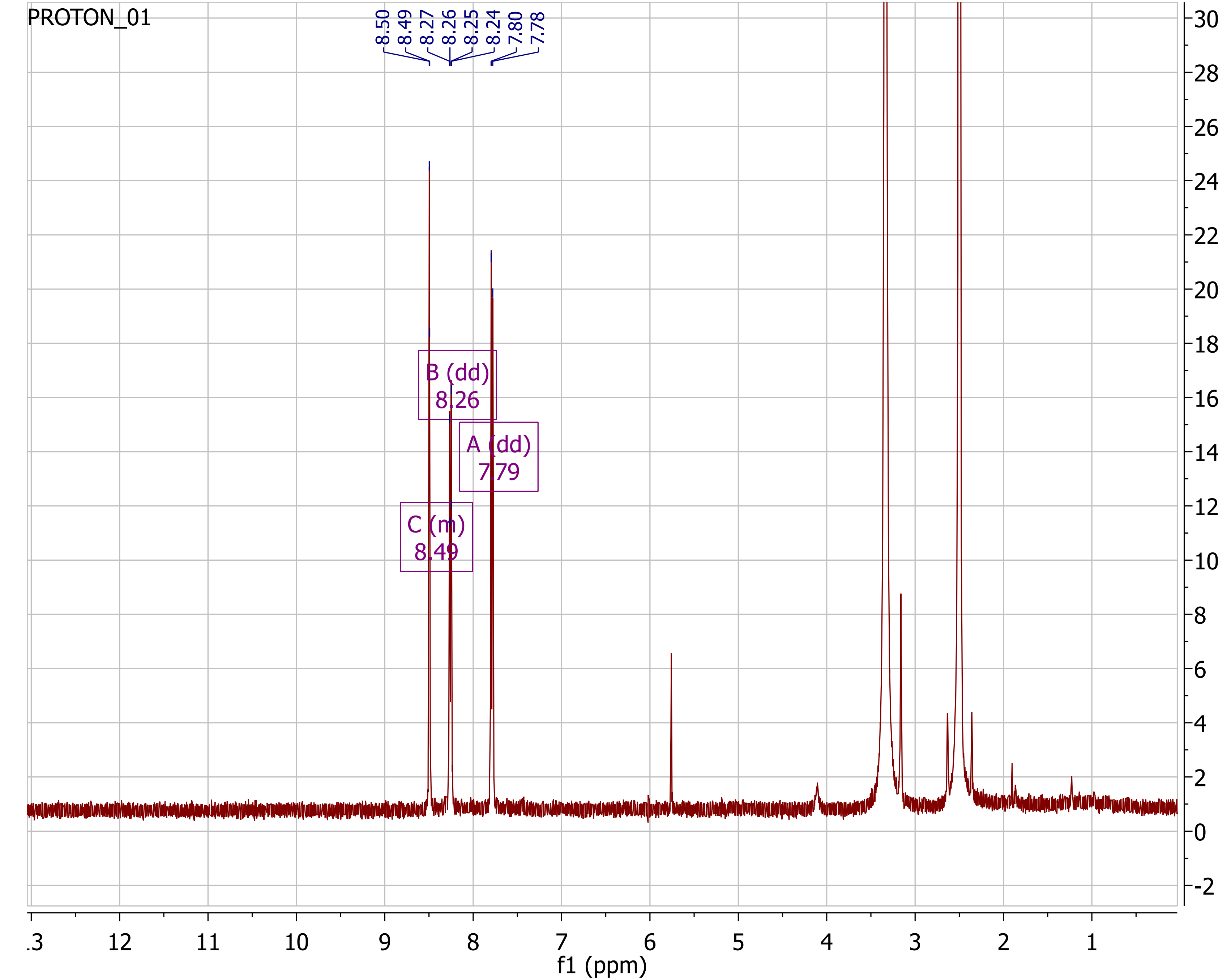


Sussex Drug Discovery Centre

| | Parameter | Value |
|---|---|---|
| 1 | Data File Name | Y:/ walkup/ sew/ 20171004/ N2118-107_01/ PROTON_01.fid/ fid |
| 2 | Title | PROTON_01 |
| 3 | Comment | |
| 4 | Origin | Varian |
| 5 | Owner | |
| 6 | Site | |
| 7 | Spectrometer | vnmrs |
| 8 | Author | |
| 9 | Solvent | dmso |
| 10 | Temperature | 30.0 |
| 11 | Pulse Sequence | s2pul |
| 12 | Experiment | 1D |
| 13 | Probe | P8898_walkup |
| 14 | Number of Scans | 8 |
| 15 | Receiver Gain | 46 |
| 16 | Relaxation Delay | 1.0000 |
| 17 | Pulse Width | 4.3000 |
| 18 | Presaturation Frequency | |
| 19 | Acquisition Time | 2.0447 |
| 20 | Acquisition Date | 2017-10-04T08:28:49 |
| 21 | Modification Date | 2017-10-04T08:29:19 |
| 22 | Class | |
| 23 | Spectrometer Frequency | 499.91 |
| 24 | Spectral Width | 8012.8 |
| 25 | Lowest Frequency | -996.5 |
| 26 | Nucleus | 1H |

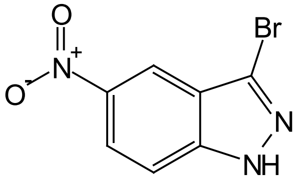


$^1$H NMR (500 MHz, DMSO-$d_6$) δ 8.53 – 8.45 (m, 1H), 8.26 (dd, $J$ = 9.1, 2.4 Hz, 1H), 7.79 (dd, $J$ = 9.2 Hz, 0.5 Hz, 1H).

Sussex Drug Discovery Centre

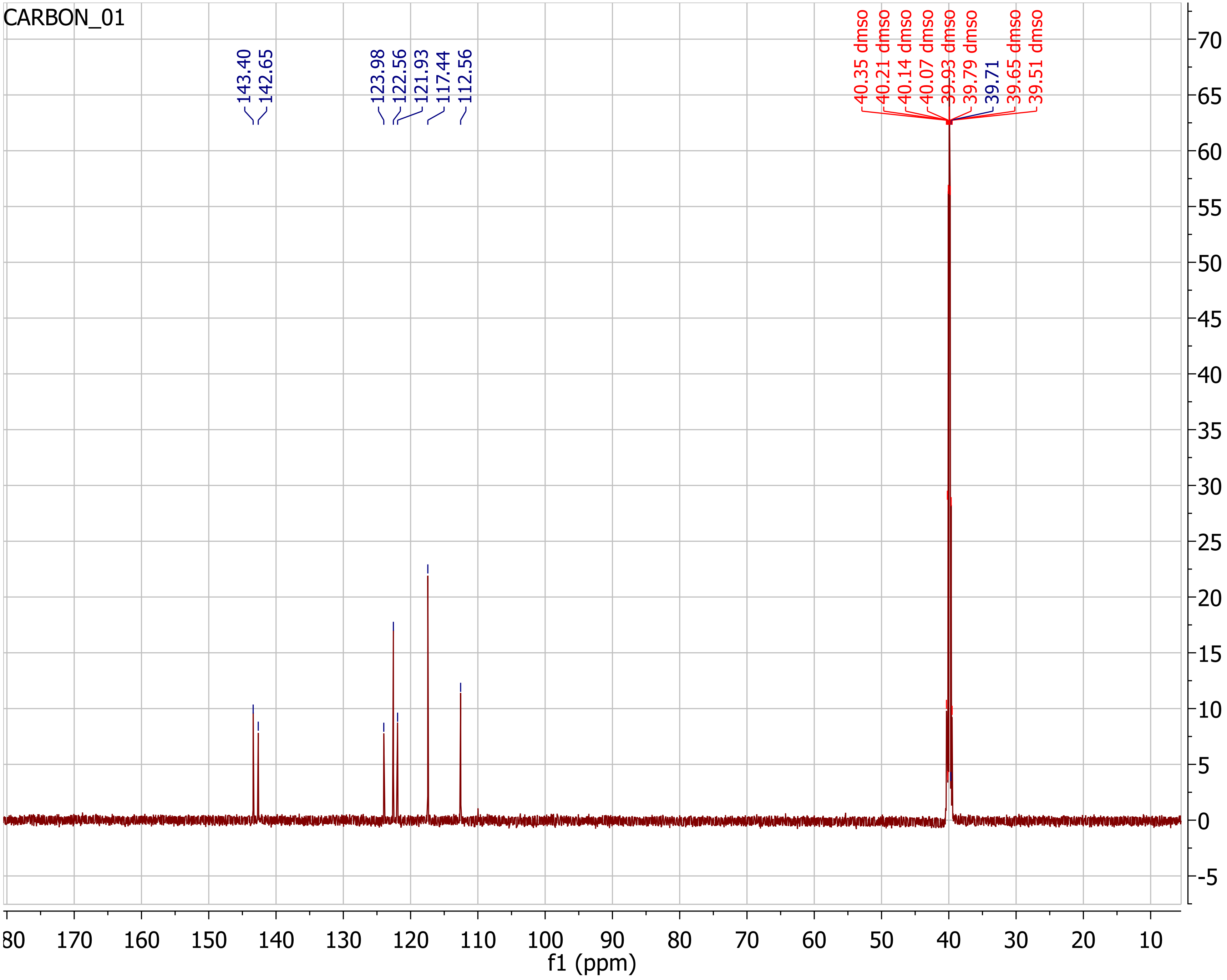


| | Parameter | Value |
|---|---|---|
| 1 | Data File Name | F:/ DB Project/ NMR/ N2118-107_01/ CARBON_01.fid/ fid |
| 2 | Title | CARBON_01 |
| 3 | Comment | |
| 4 | Origin | Varian |
| 5 | Owner | |
| 6 | Site | |
| 7 | Spectrometer | vnmrs |
| 8 | Author | |
| 9 | Solvent | dmso |
| 10 | Temperature | 25.0 |
| 11 | Pulse Sequence | s2pul |
| 12 | Experiment | 1D |
| 13 | Probe | P8891 |
| 14 | Number of Scans | 512 |
| 15 | Receiver Gain | 30 |
| 16 | Relaxation Delay | 1.0000 |
| 17 | Pulse Width | 4.1500 |
| 18 | Presaturation Frequency | |
| 19 | Acquisition Time | 0.8651 |
| 20 | Acquisition Date | 2018-01-23T18:36:17 |
| 21 | Modification Date | 2018-01-23T18:52:18 |
| 22 | Class | |
| 23 | Spectrometer Frequency | 150.81 |
| 24 | Spectral Width | 37878.8 |
| 25 | Lowest Frequency | -2414.5 |
| 26 | Nucleus | 13C |
| 27 | [illegible] | [illegible] |

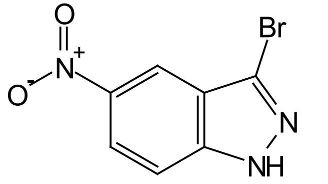

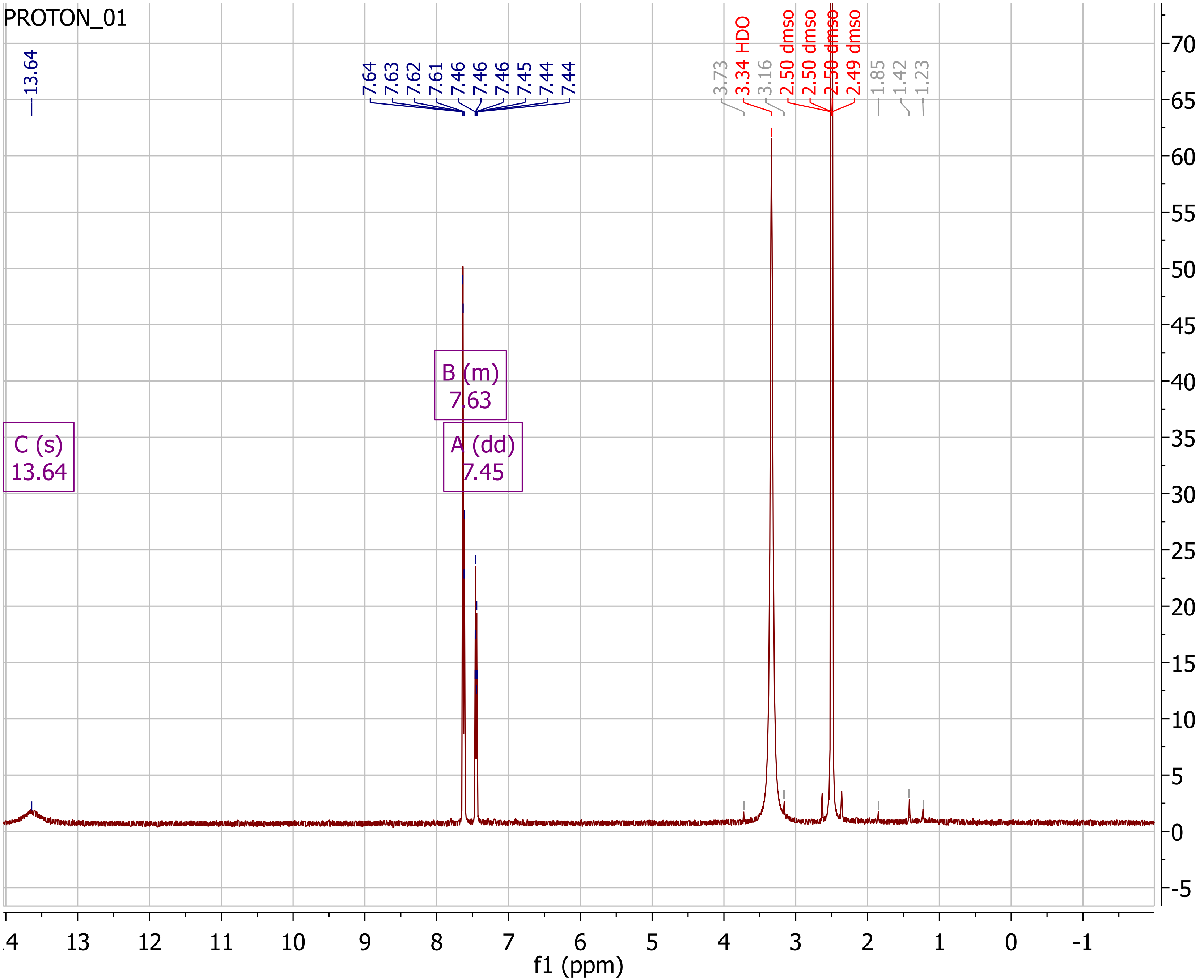


Sussex Drug Discovery Centre

| | Parameter | Value |
|---|---|---|
| 1 | Data File Name | Y:/ walkup/ sew/ 20171004/ N2118-106_01/ PROTON_01.fid/ fid |
| 2 | Title | PROTON_01 |
| 3 | Comment | |
| 4 | Origin | Varian |
| 5 | Owner | |
| 6 | Site | |
| 7 | Spectrometer | vnmrs |
| 8 | Author | |
| 9 | Solvent | dmso |
| 10 | Temperature | 30.0 |
| 11 | Pulse Sequence | s2pul |
| 12 | Experiment | 1D |
| 13 | Probe | P8898_walkup |
| 14 | Number of Scans | 8 |
| 15 | Receiver Gain | 44 |
| 16 | Relaxation Delay | 1.0000 |
| 17 | Pulse Width | 4.3000 |
| 18 | Presaturation Frequency | |
| 19 | Acquisition Time | 2.0447 |
| 20 | Acquisition Date | 2017-10-04T08:24:35 |
| 21 | Modification Date | 2017-10-04T08:25:05 |
| 22 | Class | |
| 23 | Spectrometer Frequency | 499.91 |
| 24 | Spectral Width | 8012.8 |
| 25 | Lowest Frequency | -996.5 |
| 26 | Nucleus | 1H |

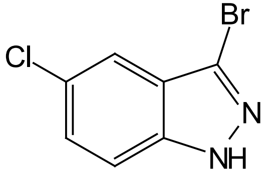


$^{1}$H NMR (500 MHz, DMSO-$d_6$) δ 13.64 (s, 1H), 7.66 – 7.59 (m, 2H), 7.45 (dd, *J* = 8.9, 2.0 Hz, 1H).

Sussex Drug Discovery Centre

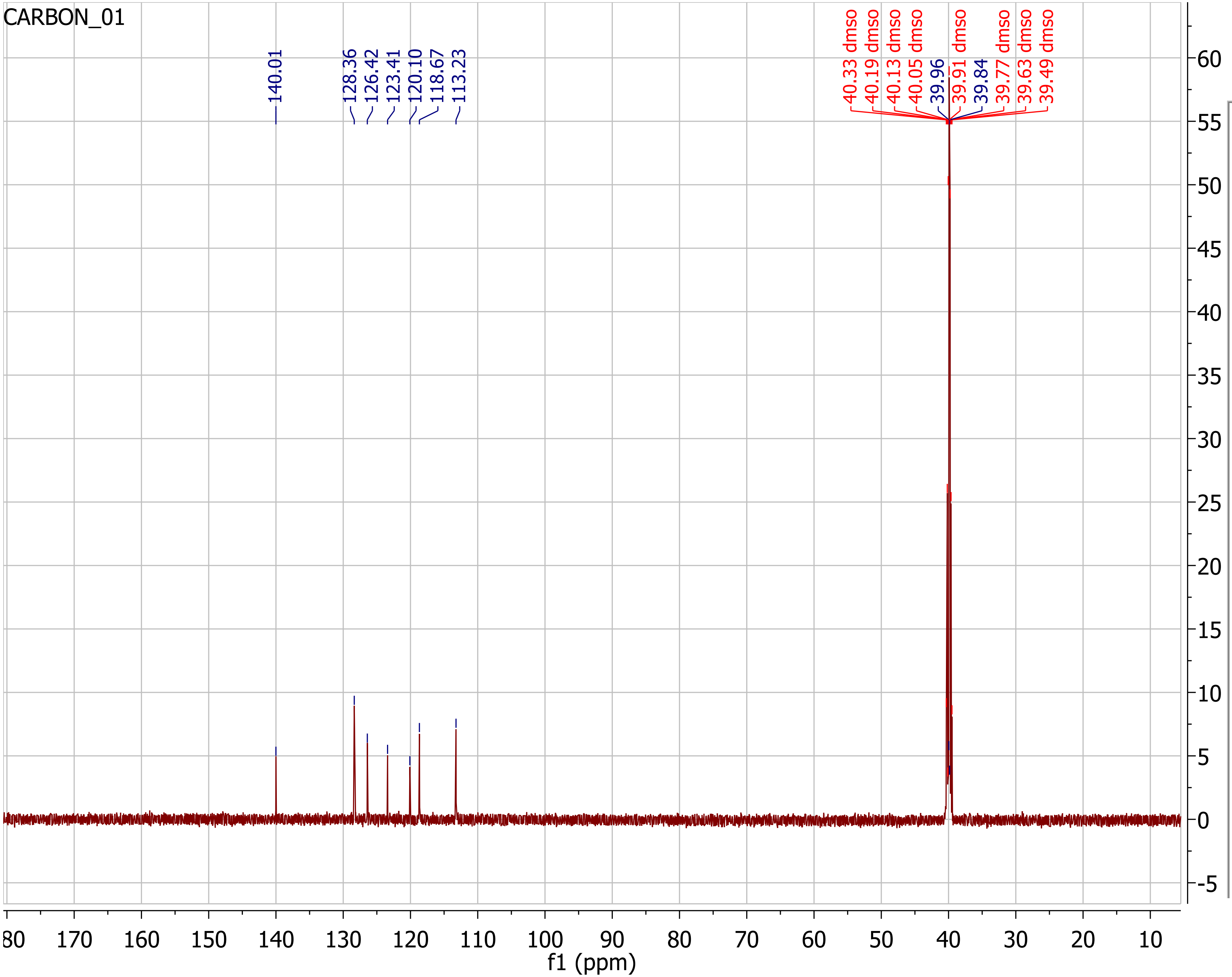


| | Parameter | Value |
|---|---|---|
| 1 | Data File Name | F:/ DB Project/ NMR/ N2118-106_01/ CARBON_01.fid/ fid |
| 2 | Title | CARBON_01 |
| 3 | Comment | |
| 4 | Origin | Varian |
| 5 | Owner | |
| 6 | Site | |
| 7 | Spectrometer | vnmrs |
| 8 | Author | |
| 9 | Solvent | dmso |
| 10 | Temperature | 25.0 |
| 11 | Pulse Sequence | s2pul |
| 12 | Experiment | 1D |
| 13 | Probe | P8891 |
| 14 | Number of Scans | 512 |
| 15 | Receiver Gain | 30 |
| 16 | Relaxation Delay | 1.0000 |
| 17 | Pulse Width | 4.1500 |
| 18 | Presaturation Frequency | |
| 19 | Acquisition Time | 0.8651 |
| 20 | Acquisition Date | 2018-01-23T19:33:20 |
| 21 | Modification Date | 2018-01-23T19:49:22 |
| 22 | Class | |
| 23 | Spectrometer Frequency | 150.81 |
| 24 | Spectral Width | 37878.8 |
| 25 | Lowest Frequency | -2410.9 |
| 26 | Nucleus | 13C |
| 27 | [illegible] | [illegible] |

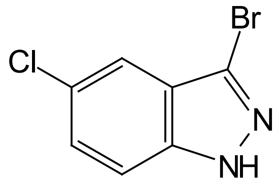

Sussex Drug Discovery Centre

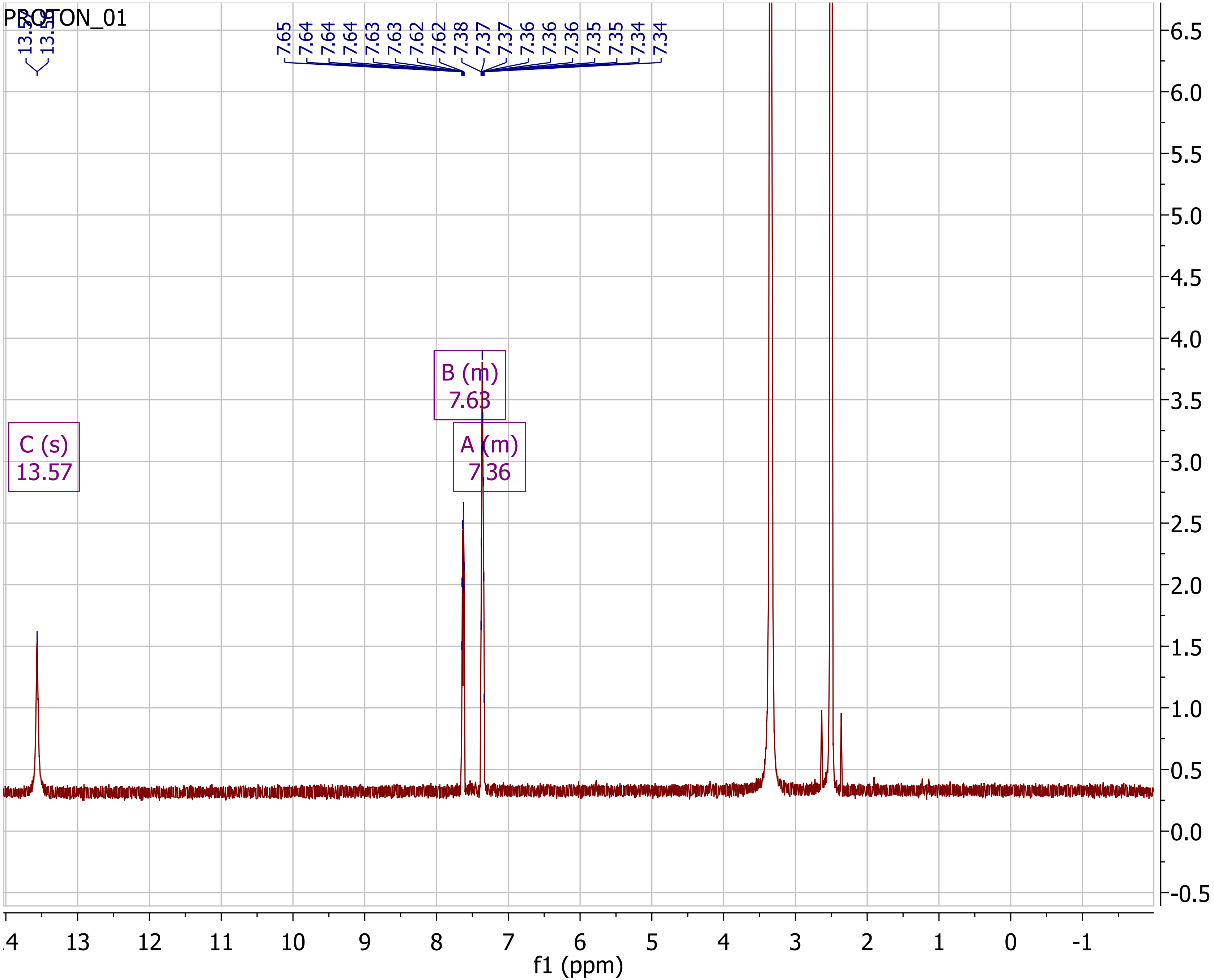


| | Parameter | Value |
|---|---|---|
| 1 | Data File Name | Y:/ walkup/ sew/ 20171103/ N2118-134_01/ PROTON_01.fid/ fid |
| 2 | Title | PROTON_01 |
| 3 | Comment | |
| 4 | Origin | Varian |
| 5 | Owner | |
| 6 | Site | |
| 7 | Spectrometer | vnmrs |
| 8 | Author | |
| 9 | Solvent | dmso |
| 10 | Temperature | 30.0 |
| 11 | Pulse Sequence | s2pul |
| 12 | Experiment | 1D |
| 13 | Probe | P8898_walkup |
| 14 | Number of Scans | 8 |
| 15 | Receiver Gain | 30 |
| 16 | Relaxation Delay | 1.0000 |
| 17 | Pulse Width | 4.3000 |
| 18 | Presaturation Frequency | |
| 19 | Acquisition Time | 2.0447 |
| 20 | Acquisition Date | 2017-11-03T16:01:02 |
| 21 | Modification Date | 2017-11-03T16:01:32 |
| 22 | Class | |
| 23 | Spectrometer Frequency | 499.91 |
| 24 | Spectral Width | 8012.8 |
| 25 | Lowest Frequency | -996.5 |
| 26 | Nucleus | 1H |

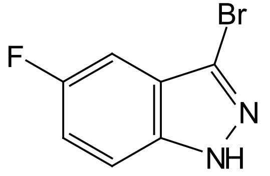


$^{1}$H NMR (500 MHz, DMSO-$d_6$) δ 13.57 (s, 1H), 7.67 – 7.58 (m, 1H), 7.39 – 7.32 (m, 2H).

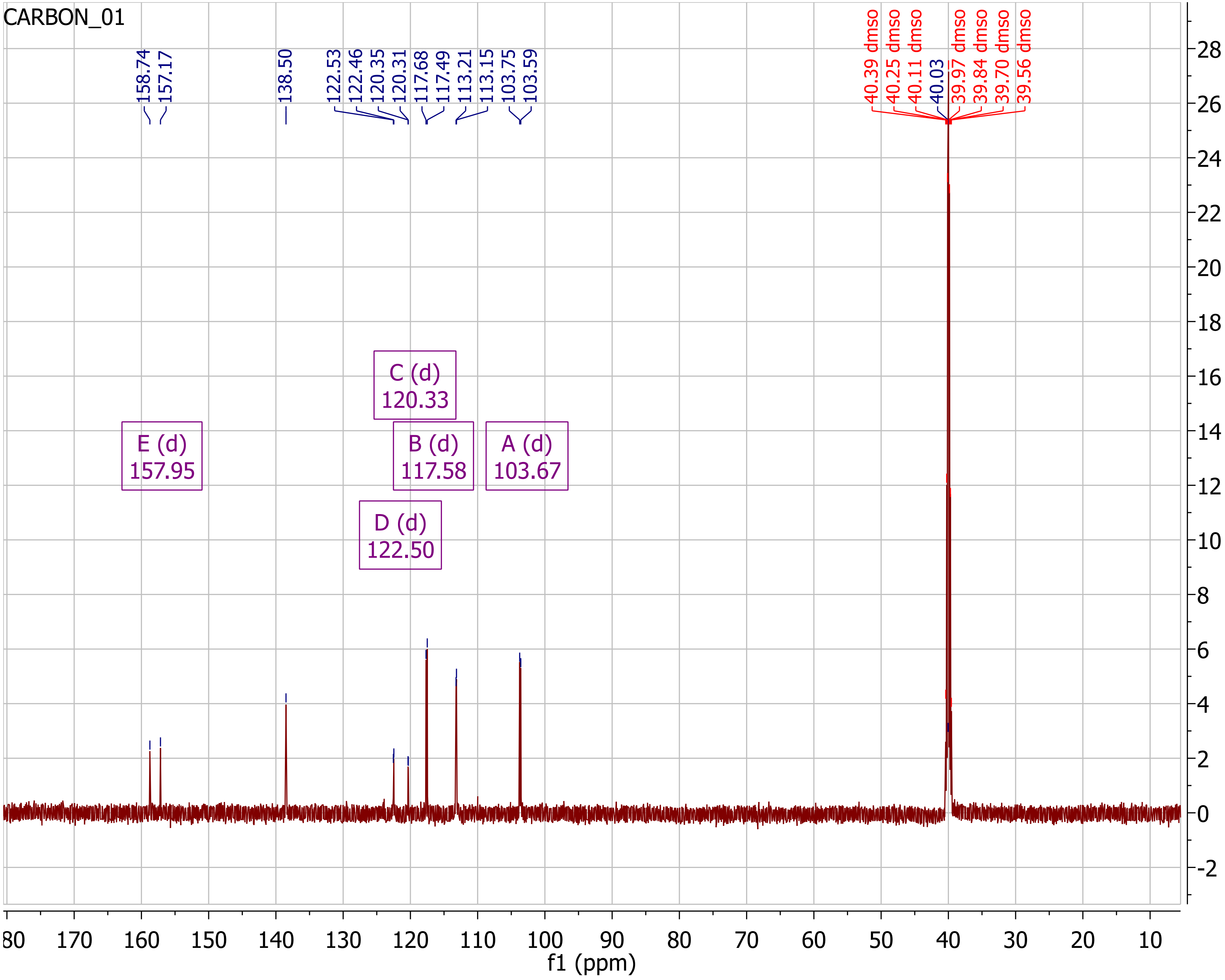


Sussex Drug Discovery Centre

| | Parameter | Value |
|---|---|---|
| 1 | Data File Name | F:/ DB Project/ NMR/ Conc C NMR/ N2118-134_01/ CARBON_01.fid/ fid |
| 2 | Title | CARBON_01 |
| 3 | Comment | |
| 4 | Origin | Varian |
| 5 | Owner | |
| 6 | Site | |
| 7 | Spectrometer | vnmrs |
| 8 | Author | |
| 9 | Solvent | dmso |
| 10 | Temperature | 25.0 |
| 11 | Pulse Sequence | s2pul |
| 12 | Experiment | 1D |
| 13 | Probe | P8891 |
| 14 | Number of Scans | 256 |
| 15 | Receiver Gain | 30 |
| 16 | Relaxation Delay | 1.0000 |
| 17 | Pulse Width | 4.1500 |
| 18 | Presaturation Frequency | |
| 19 | Acquisition Time | 0.8651 |
| 20 | Acquisition Date | 2018-01-24T10:19:11 |
| 21 | Modification Date | 2018-01-24T10:27:14 |
| 22 | Class | |
| 23 | Spectrometer Frequency | 150.81 |
| 24 | Spectral Width | 37878.8 |
| 25 | Lowest Frequency | -2420.9 |
| 26 | Nucleus | 13C |

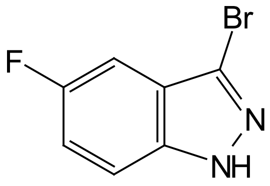


$^{13}$C NMR (151 MHz, DMSO-$d_6$) δ 157.95 (d, $J$ = 237.3 Hz), 122.50 (d, $J$ = 10.9 Hz), 120.33 (d, $J$ = 5.8 Hz), 117.58 (d, $J$ = 27.7 Hz), 103.67 (d, $J$ = 24.5 Hz).

Sussex Drug Discovery Centre

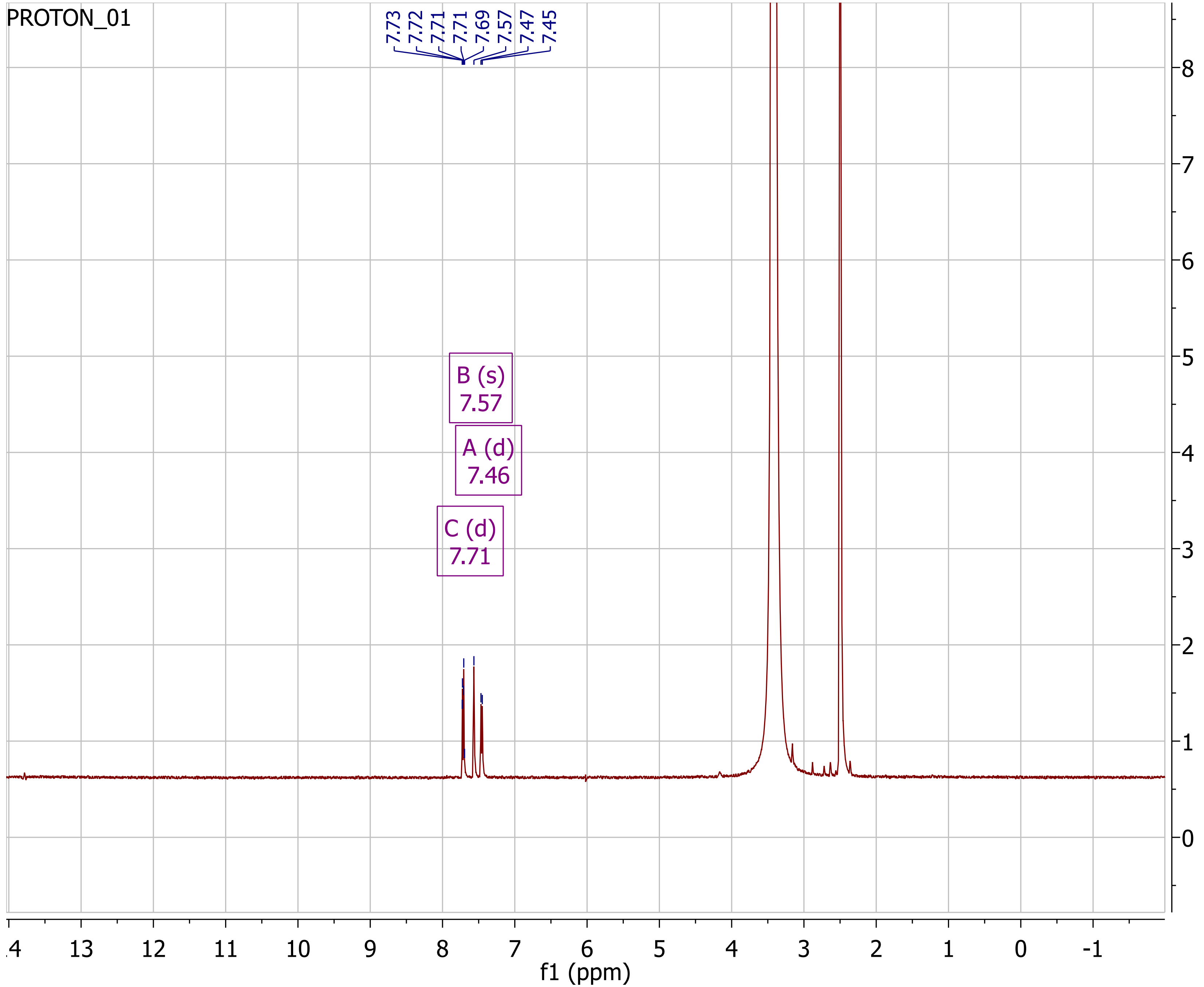


| | Parameter | Value |
|---|---|---|
| 1 | Data File Name | Y:/ walkup/ sew/ 20171103/ N2118-135_01/ PROTON_01.fid/ fid |
| 2 | Title | PROTON_01 |
| 3 | Comment | |
| 4 | Origin | Varian |
| 5 | Owner | |
| 6 | Site | |
| 7 | Spectrometer | vnmrs |
| 8 | Author | |
| 9 | Solvent | dmso |
| 10 | Temperature | 30.0 |
| 11 | Pulse Sequence | s2pul |
| 12 | Experiment | 1D |
| 13 | Probe | P8898_walkup |
| 14 | Number of Scans | 8 |
| 15 | Receiver Gain | 18 |
| 16 | Relaxation Delay | 1.0000 |
| 17 | Pulse Width | 4.3000 |
| 18 | Presaturation Frequency | |
| 19 | Acquisition Time | 2.0447 |
| 20 | Acquisition Date | 2017-11-03T16:04:53 |
| 21 | Modification Date | 2017-11-03T16:06:23 |
| 22 | Class | |
| 23 | Spectrometer Frequency | 499.91 |
| 24 | Spectral Width | 8012.8 |
| 25 | Lowest Frequency | -996.5 |
| 26 | Nucleus | 1H |

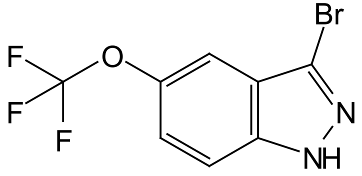


$^{1}$H NMR (500 MHz, DMSO-$d_6$) δ 7.71 (d, $J$ = 8.9 Hz, 1H), 7.57 (s, 1H), 7.46 (d, $J$ = 9.0 Hz, 1H).

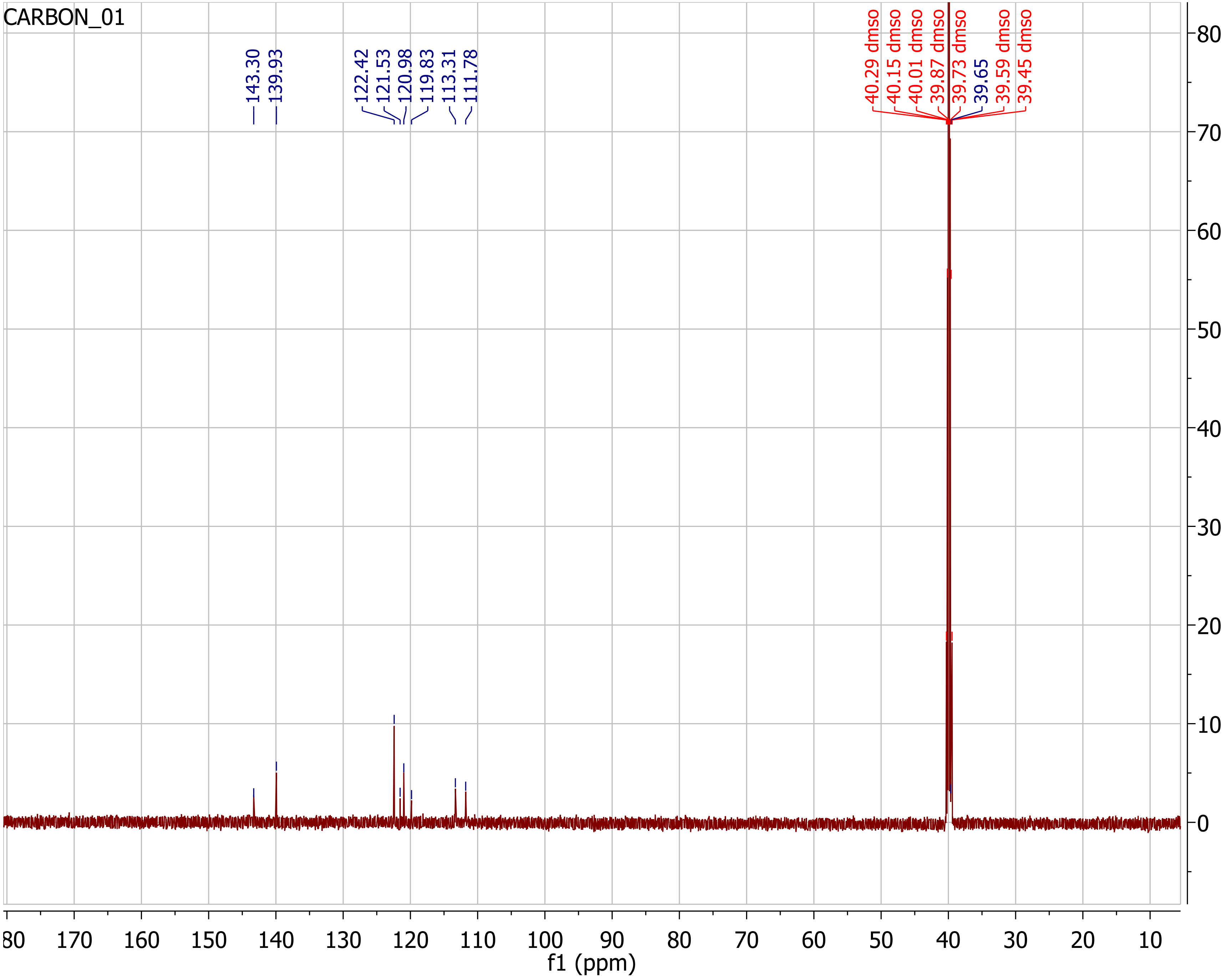


Sussex Drug Discovery Centre

| | Parameter | Value |
|---|---|---|
| 1 | Data File Name | F:/ DB Project/ NMR/ N2118-135_01/ CARBON_01.fid/ fid |
| 2 | Title | CARBON_01 |
| 3 | Comment | |
| 4 | Origin | Varian |
| 5 | Owner | |
| 6 | Site | |
| 7 | Spectrometer | vnmrs |
| 8 | Author | |
| 9 | Solvent | dmso |
| 10 | Temperature | 25.0 |
| 11 | Pulse Sequence | s2pul |
| 12 | Experiment | 1D |
| 13 | Probe | P8891 |
| 14 | Number of Scans | 1000 |
| 15 | Receiver Gain | 30 |
| 16 | Relaxation Delay | 1.0000 |
| 17 | Pulse Width | 4.1500 |
| 18 | Presaturation Frequency | |
| 19 | Acquisition Time | 0.8651 |
| 20 | Acquisition Date | 2018-01-24T03:35:06 |
| 21 | Modification Date | 2018-01-24T04:06:24 |
| 22 | Class | |
| 23 | Spectrometer Frequency | 150.81 |
| 24 | Spectral Width | 37878.8 |
| 25 | Lowest Frequency | -2405.2 |
| 26 | Nucleus | 13C |
| [illegible] | [illegible] | [illegible] |

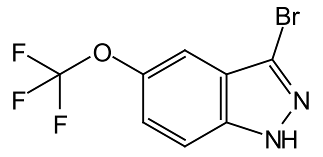

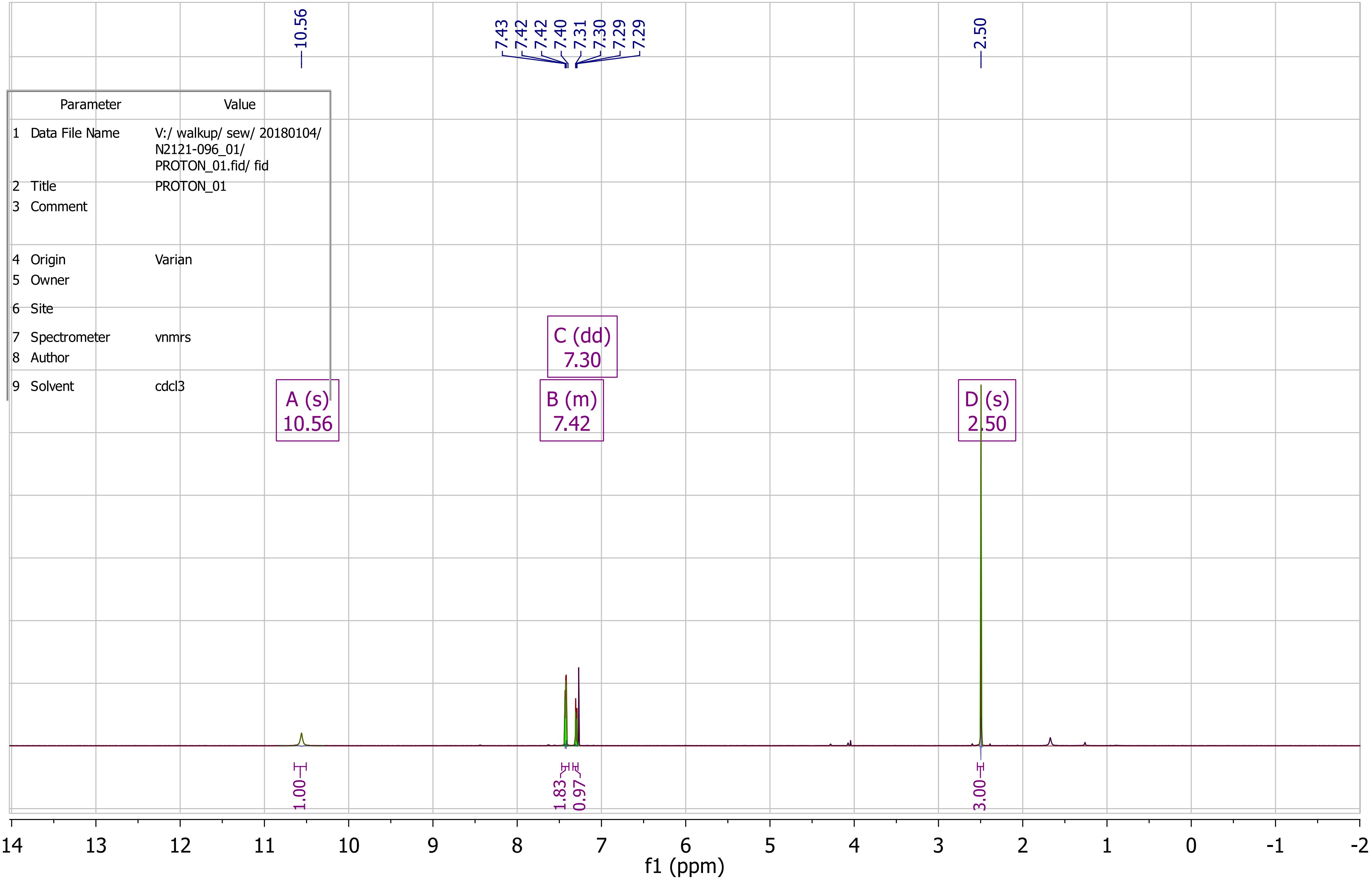


$^{1}$H NMR (600 MHz, Chloroform-*d*) δ 10.56 (s, 1H), 7.47 – 7.39 (m, 2H), 7.30 (dd, *J* = 8.7, 1.4 Hz, 1H), 2.50 (s, 3H).

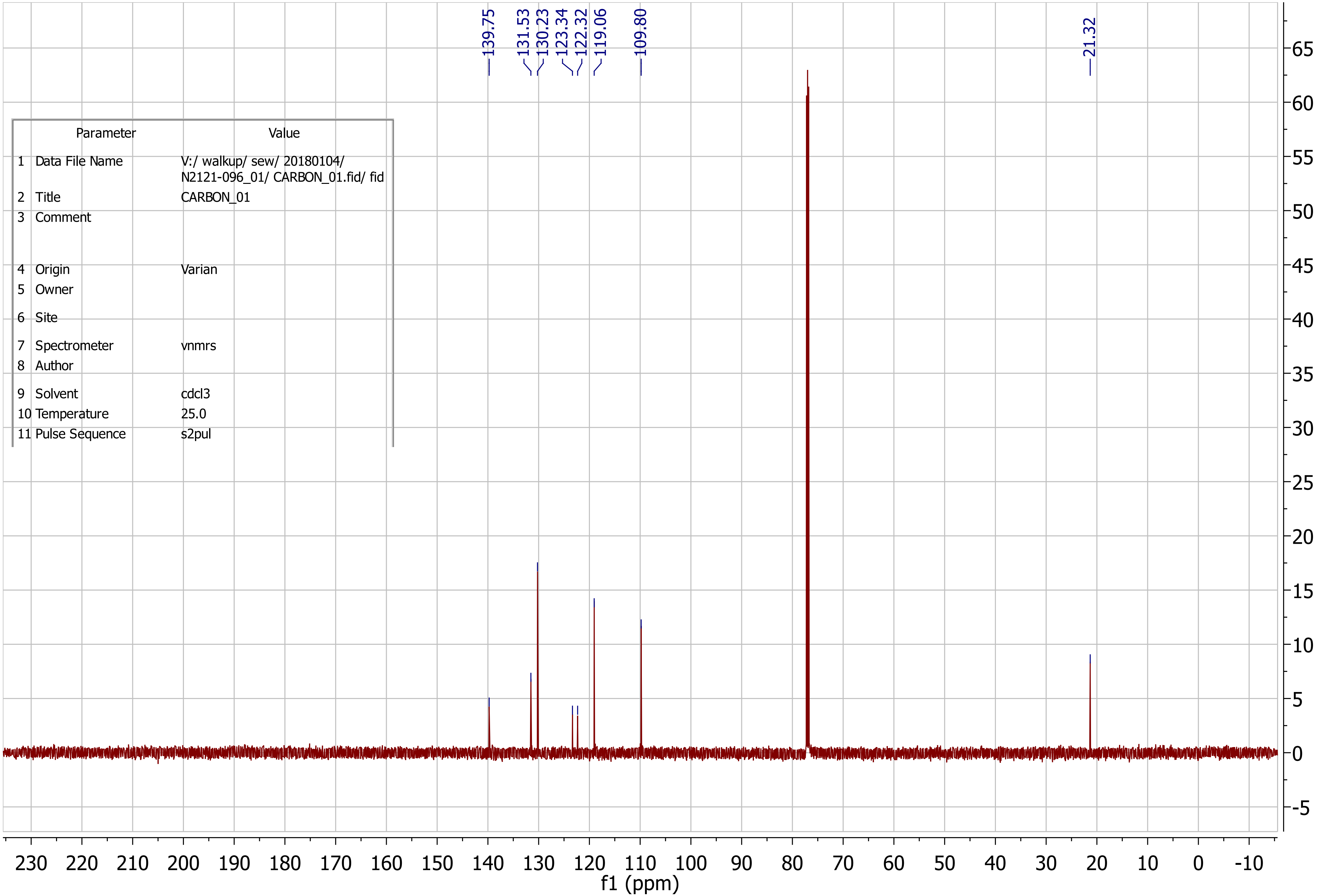


$^{13}$C NMR (151 MHz, cdcl$_3$) δ 139.75, 131.53, 130.23, 123.34, 122.32, 119.06, 109.80, 21.32.

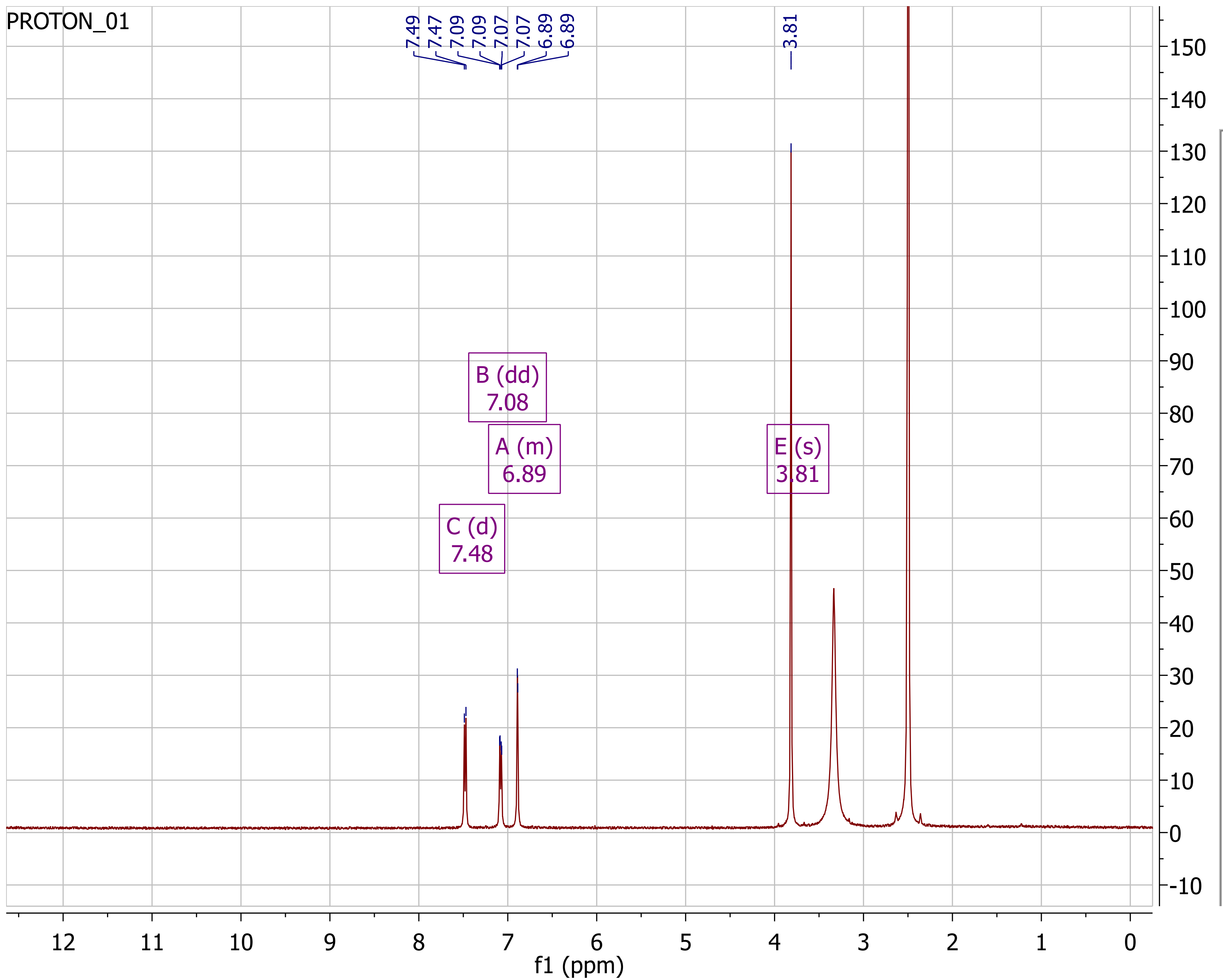


Sussex Drug Discovery Centre

| | Parameter | Value |
|---|---|---|
| 1 | Data File Name | Y:/ walkup/ sew/ 20171004/ N2118-105_01/ PROTON_01.fid/ fid |
| 2 | Title | PROTON_01 |
| 3 | Comment | |
| 4 | Origin | Varian |
| 5 | Owner | |
| 6 | Site | |
| 7 | Spectrometer | vnmrs |
| 8 | Author | |
| 9 | Solvent | dmso |
| 10 | Temperature | 30.0 |
| 11 | Pulse Sequence | s2pul |
| 12 | Experiment | 1D |
| 13 | Probe | P8898_walkup |
| 14 | Number of Scans | 8 |
| 15 | Receiver Gain | 44 |
| 16 | Relaxation Delay | 1.0000 |
| 17 | Pulse Width | 4.3000 |
| 18 | Presaturation Frequency | |
| 19 | Acquisition Time | 2.0447 |
| 20 | Acquisition Date | 2017-10-04T08:19:30 |
| 21 | Modification Date | 2017-10-04T08:20:07 |
| 22 | Class | |
| 23 | Spectrometer Frequency | 499.91 |
| 24 | Spectral Width | 8012.8 |
| 25 | Lowest Frequency | -996.5 |
| 26 | Nucleus | 1H |

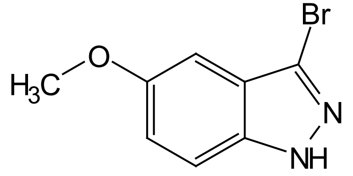


$^{1}$H NMR (500 MHz, DMSO-$d_6$) δ 13.29 (s, 1H), 7.48 (d, $J$ = 9.0 Hz, 1H), 7.08 (dd, $J$ = 9.0, 2.5 Hz, 1H), 6.95 – 6.82 (m, 1H), 3.81 (s, 3H).

CARBON_01

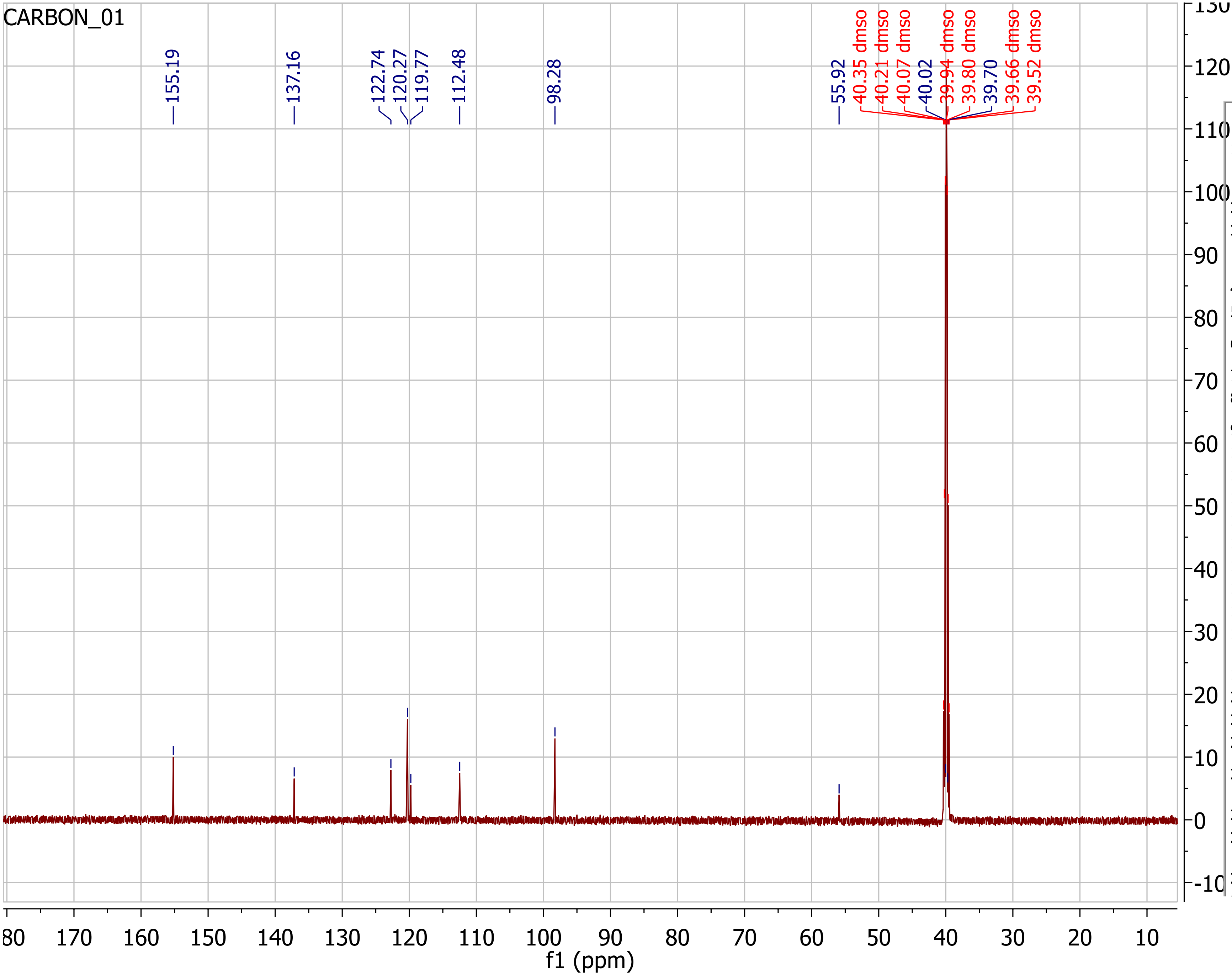


Sussex Drug Discovery Centre

| | Parameter | Value |
|---|---|---|
| 1 | Data File Name | F:/ DB Project/ NMR/ N2118-105_01/ CARBON_01.fid/ fid |
| 2 | Title | CARBON_01 |
| 3 | Comment | |
| 4 | Origin | Varian |
| 5 | Owner | |
| 6 | Site | |
| 7 | Spectrometer | vnmrs |
| 8 | Author | |
| 9 | Solvent | dmso |
| 10 | Temperature | 25.0 |
| 11 | Pulse Sequence | s2pul |
| 12 | Experiment | 1D |
| 13 | Probe | P8891 |
| 14 | Number of Scans | 1000 |
| 15 | Receiver Gain | 30 |
| 16 | Relaxation Delay | 1.0000 |
| 17 | Pulse Width | 4.1500 |
| 18 | Presaturation Frequency | |
| 19 | Acquisition Time | 0.8651 |
| 20 | Acquisition Date | 2018-01-24T04:19:28 |
| 21 | Modification Date | 2018-01-24T04:50:42 |
| 22 | Class | |
| 23 | Spectrometer Frequency | 150.81 |
| 24 | Spectral Width | 37878.8 |
| 25 | Lowest Frequency | -2415.0 |
| 26 | Nucleus | 13C |
| 27 | [illegible] | [illegible] |

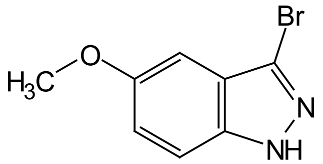

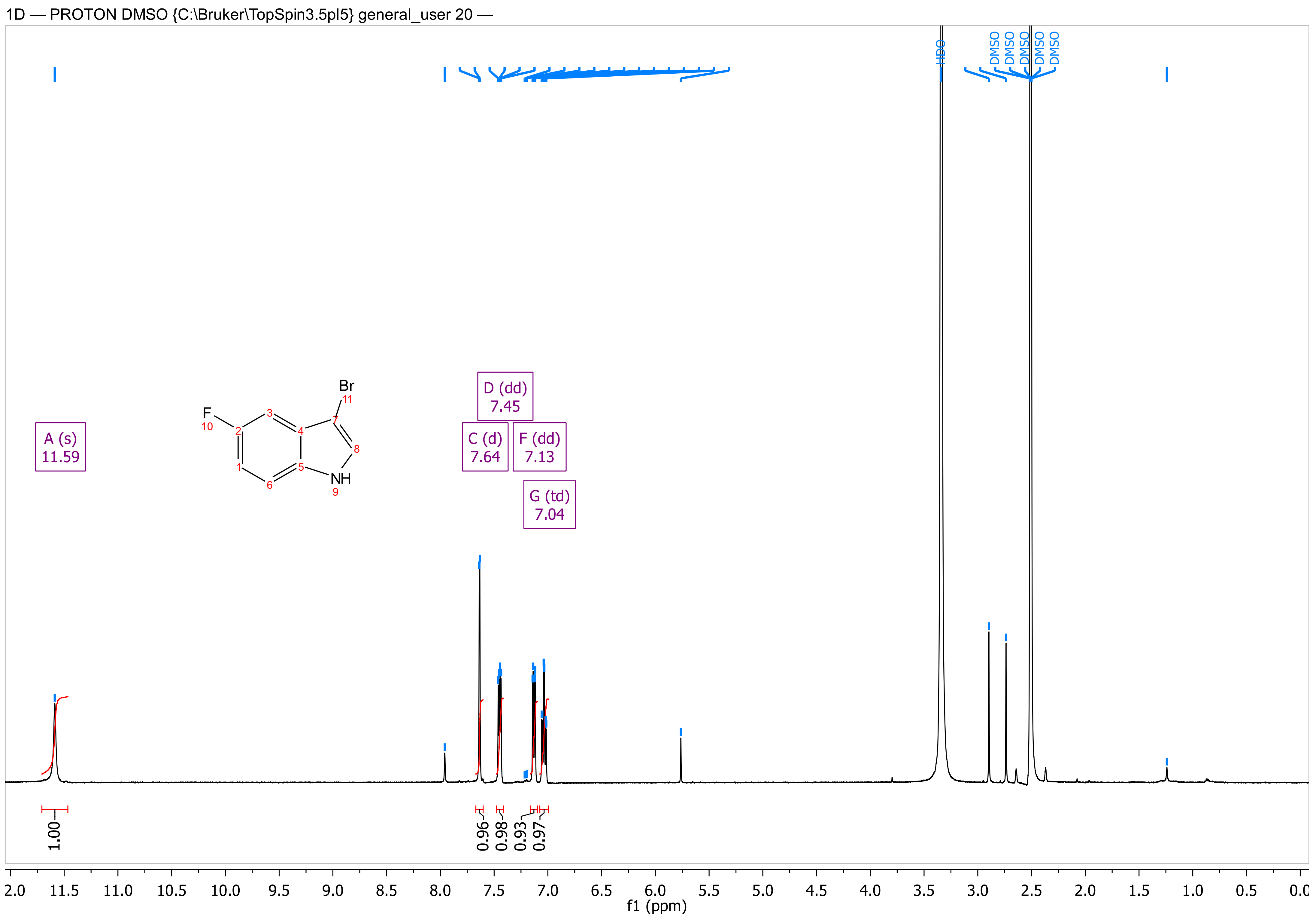
1D — PROTON DMSO {C:\Bruker\TopSpin3.5pl5} general_user 20 —
HDO
DMSO
DMSO
DMSO
DMSO
DMSO
A (s)
11.59
Br
F
NH
D (dd)
7.45
C (d)
7.64
F (dd)
7.13
G (td)
7.04
1.00
0.96
0.98
0.93
0.97
f1 (ppm)

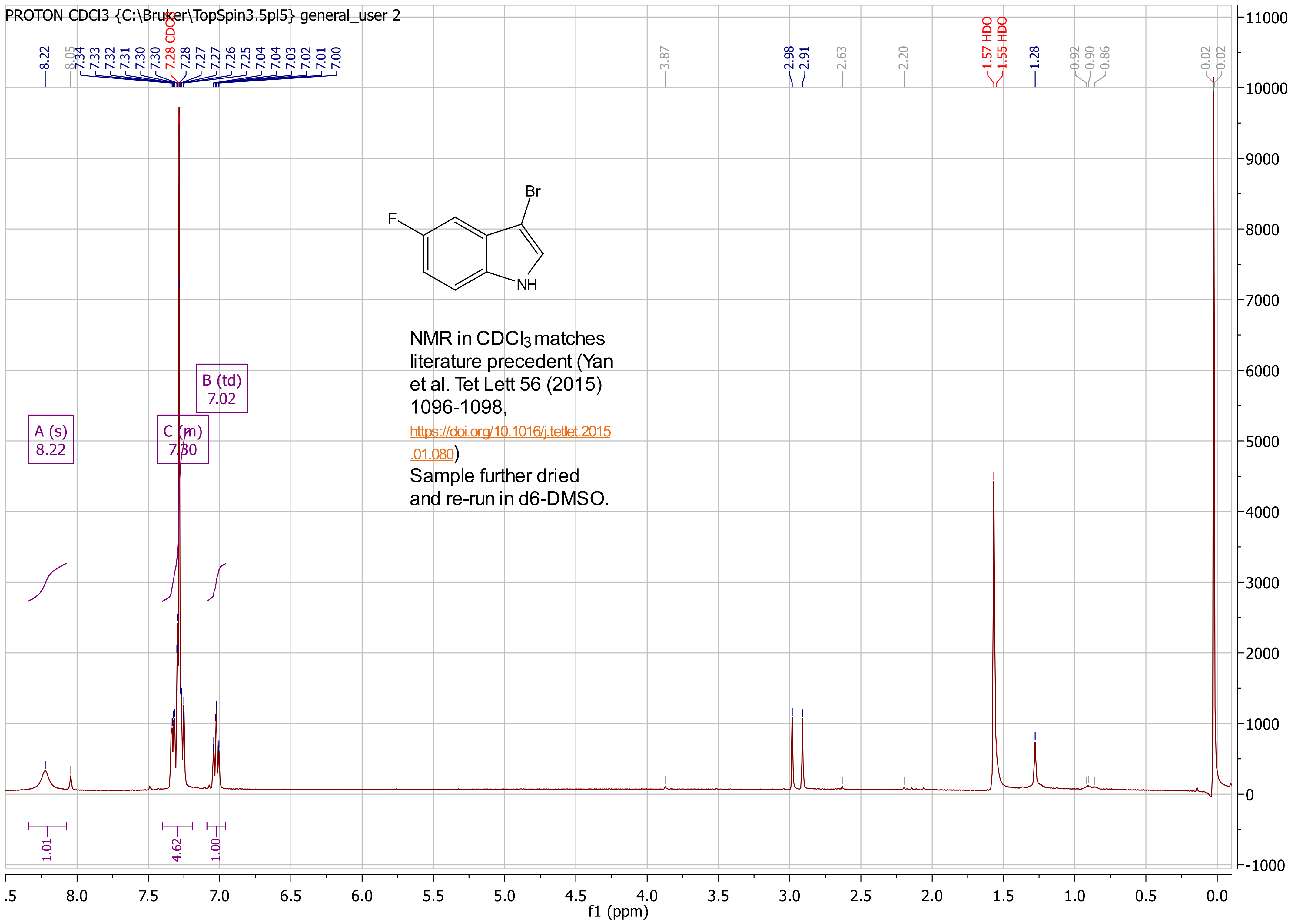
PROTON CDCl3 {C:\Bruker\TopSpin3.5pl5} general_user 2
8.22
8.05
7.34
7.33
7.32
7.31
7.30
7.30
7.28 CDCl3
7.28
7.27
7.27
7.26
7.25
7.04
7.04
7.03
7.02
7.01
7.00
3.87
2.98
2.91
2.63
2.20
1.57 HDO
1.55 HDO
1.28
0.92
0.90
0.86
0.02
-0.02
Br
F
NH
NMR in $CDCl_3$ matches literature precedent (Yan et al. Tet Lett 56 (2015) 1096-1098, https://doi.org/10.1016/j.tetlet.2015.01.080)
Sample further dried and re-run in d6-DMSO.
A (s)
8.22
B (td)
7.02
C (m)
7.30
1.01
4.62
1.00
f1 (ppm)

1D — PROTON DMSO {C:\Bruker\TopSpin3.5pl5} general_user 21 —

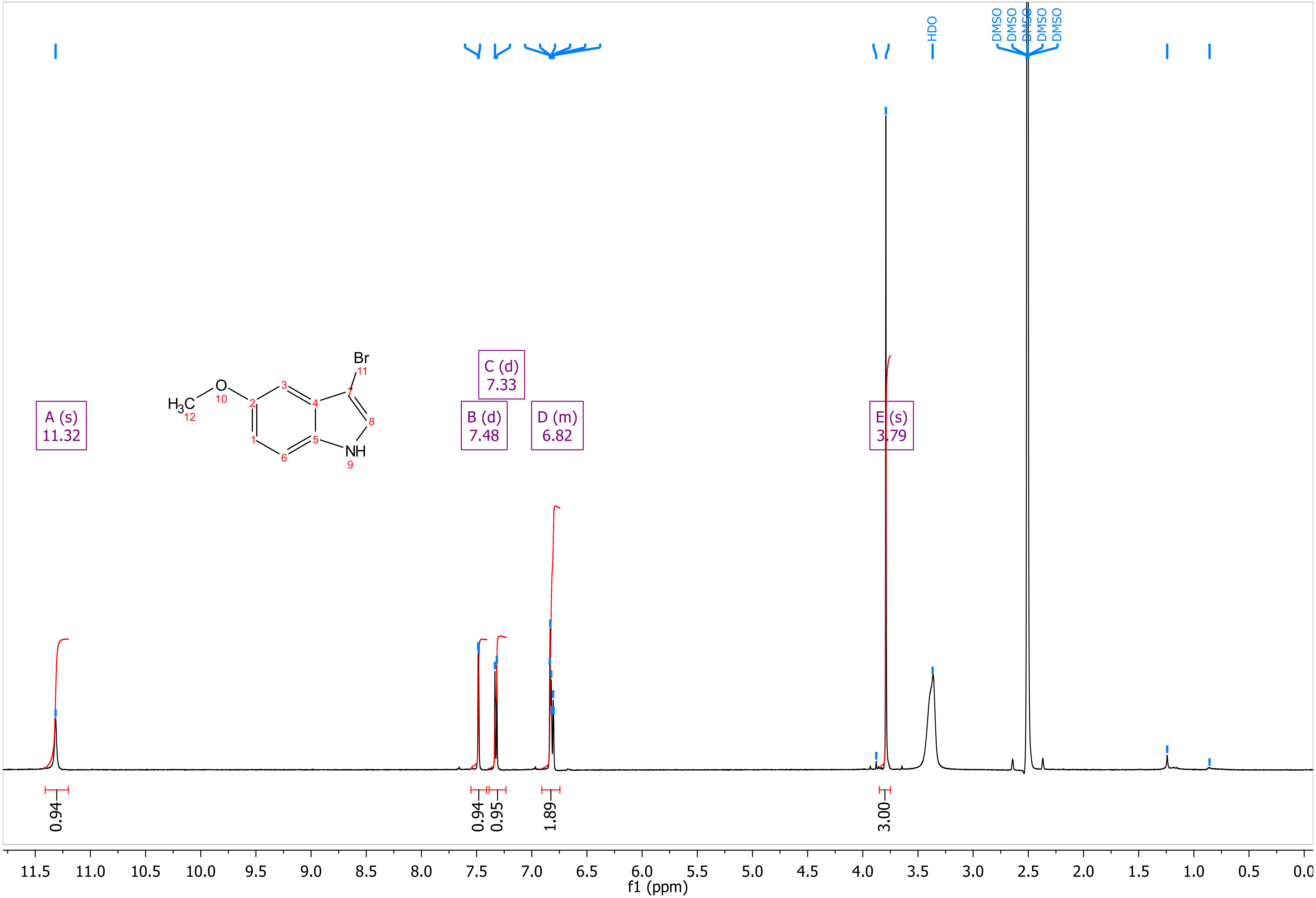

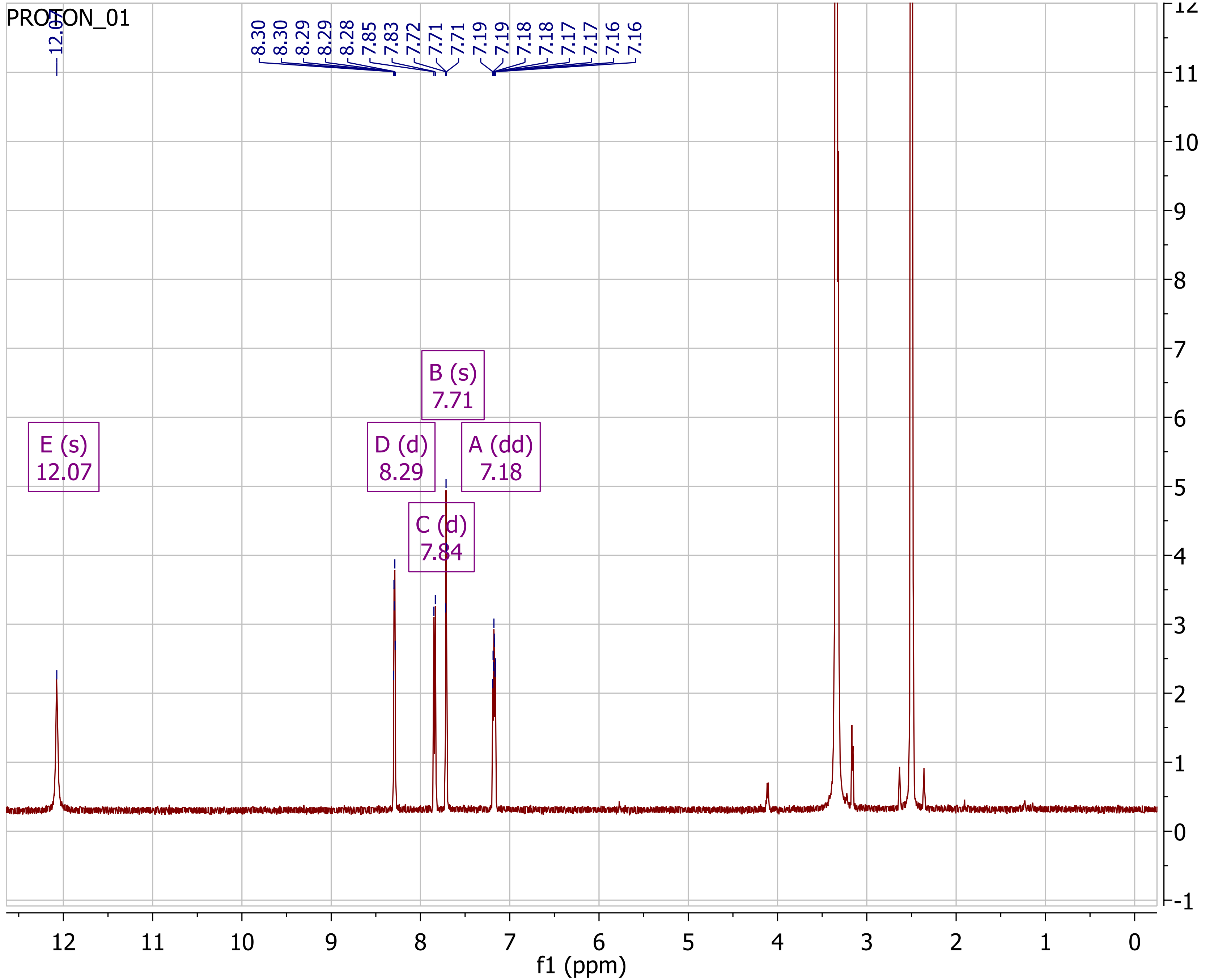


Sussex Drug Discovery Centre

| | Parameter | Value |
|---|---|---|
| 1 | Data File Name | Y:/ walkup/ sew/ 20171026/ N2118-128_01/ PROTON_01.fid/ fid |
| 2 | Title | PROTON_01 |
| 3 | Comment | |
| 4 | Origin | Varian |
| 5 | Owner | |
| 6 | Site | |
| 7 | Spectrometer | vnmrs |
| 8 | Author | |
| 9 | Solvent | dmso |
| 10 | Temperature | 30.0 |
| 11 | Pulse Sequence | s2pul |
| 12 | Experiment | 1D |
| 13 | Probe | P8898_walkup |
| 14 | Number of Scans | 8 |
| 15 | Receiver Gain | 30 |
| 16 | Relaxation Delay | 1.0000 |
| 17 | Pulse Width | 4.3000 |
| 18 | Presaturation Frequency | |
| 19 | Acquisition Time | 2.0447 |
| 20 | Acquisition Date | 2017-10-26T11:00:08 |
| 21 | Modification Date | 2017-10-26T11:00:37 |
| 22 | Class | |
| 23 | Spectrometer Frequency | 499.91 |
| 24 | Spectral Width | 8012.8 |
| 25 | Lowest Frequency | -996.5 |
| 26 | Nucleus | 1H |

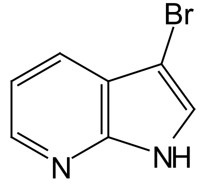


$^1$H NMR (500 MHz, DMSO-$d_6$) δ 12.07 (s, 1H), 8.29 (d, $J$ = 4.6 Hz, 1H), 7.84 (d, $J$ = 7.9 Hz, 1H), 7.71 (s, 1H), 7.18 (dd, $J$ = 7.9, 4.6 Hz, 1H).

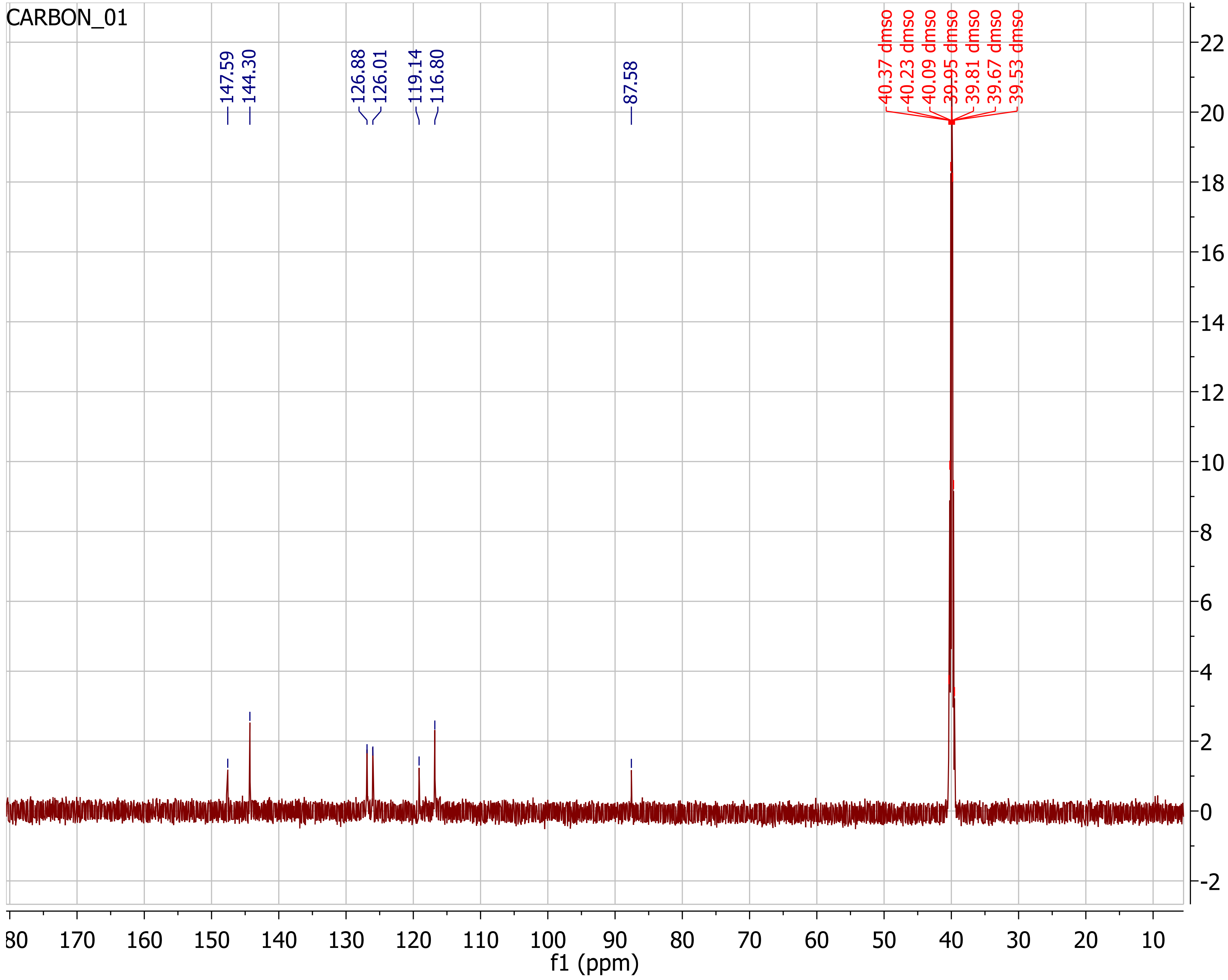


Sussex Drug Discovery Centre

| | Parameter | Value |
|---|---|---|
| 1 | Data File Name | F:/ DB Project/ NMR/ Conc C NMR/ N2118-128_02/ CARBON_01.fid/ fid |
| 2 | Title | CARBON_01 |
| 3 | Comment | |
| 4 | Origin | Varian |
| 5 | Owner | |
| 6 | Site | |
| 7 | Spectrometer | vnmrs |
| 8 | Author | |
| 9 | Solvent | dmso |
| 10 | Temperature | 25.0 |
| 11 | Pulse Sequence | s2pul |
| 12 | Experiment | 1D |
| 13 | Probe | P8891 |
| 14 | Number of Scans | 256 |
| 15 | Receiver Gain | 30 |
| 16 | Relaxation Delay | 1.0000 |
| 17 | Pulse Width | 4.1500 |
| 18 | Presaturation Frequency | |
| 19 | Acquisition Time | 0.8651 |
| 20 | Acquisition Date | 2018-01-24T10:30:53 |
| 21 | Modification Date | 2018-01-24T10:38:56 |
| 22 | Class | |
| 23 | Spectrometer Frequency | 150.81 |
| 24 | Spectral Width | 37878.8 |
| 25 | Lowest Frequency | -2417.1 |
| 26 | Nucleus | [illegible] |

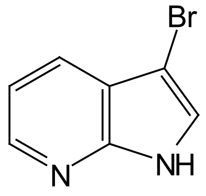

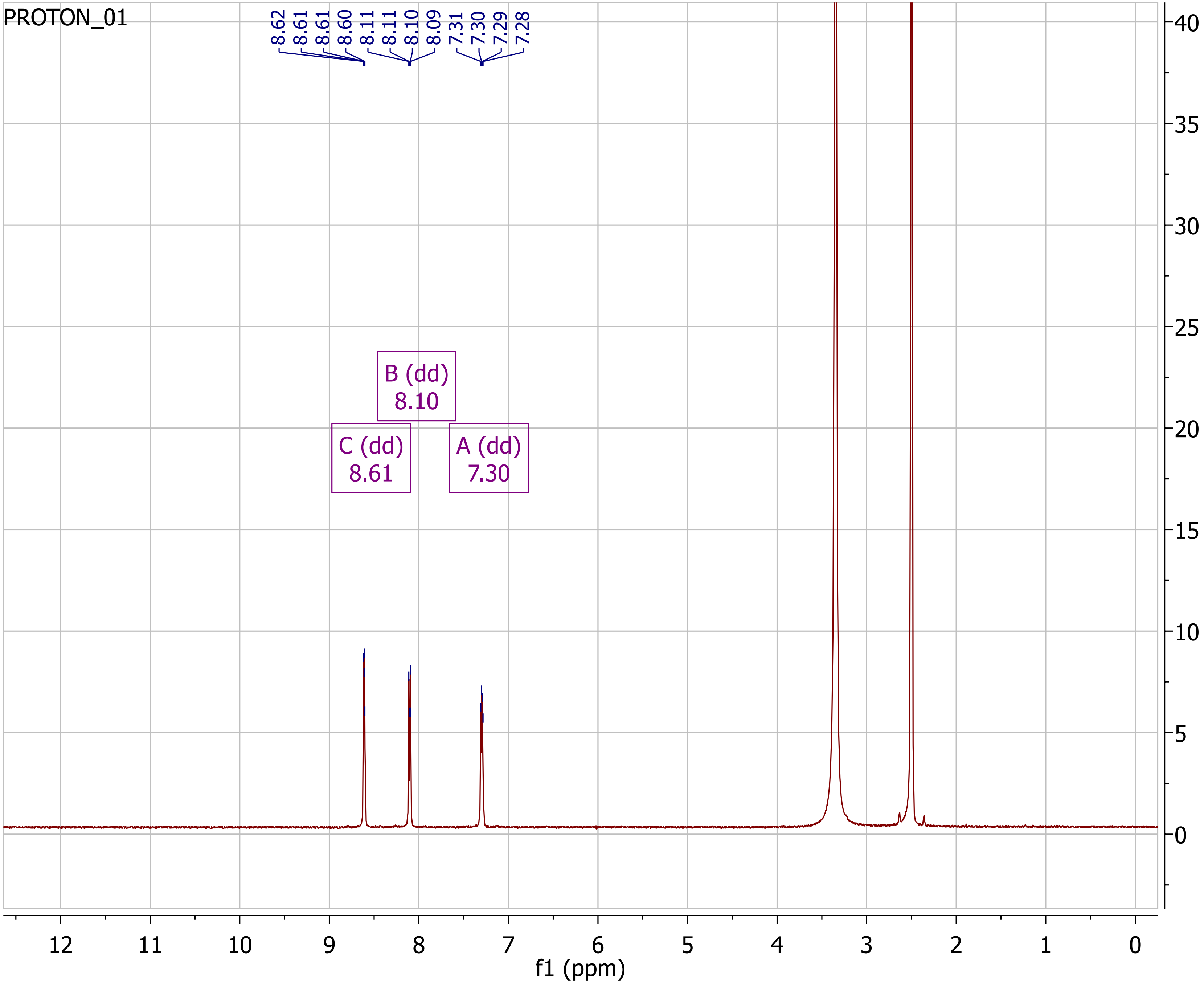


Sussex Drug Discovery Centre

| | Parameter | Value |
|---|---|---|
| 1 | Data File Name | Y:/ walkup/ sew/ 20171103/ N2118-137_01/ PROTON_01.fid/ fid |
| 2 | Title | PROTON_01 |
| 3 | Comment | |
| 4 | Origin | Varian |
| 5 | Owner | |
| 6 | Site | |
| 7 | Spectrometer | vnmrs |
| 8 | Author | |
| 9 | Solvent | dmso |
| 10 | Temperature | 30.0 |
| 11 | Pulse Sequence | s2pul |
| 12 | Experiment | 1D |
| 13 | Probe | P8898_walkup |
| 14 | Number of Scans | 8 |
| 15 | Receiver Gain | 30 |
| 16 | Relaxation Delay | 1.0000 |
| 17 | Pulse Width | 4.3000 |
| 18 | Presaturation Frequency | |
| 19 | Acquisition Time | 2.0447 |
| 20 | Acquisition Date | 2017-11-03T16:10:30 |
| 21 | Modification Date | 2017-11-03T16:11:07 |
| 22 | Class | |
| 23 | Spectrometer Frequency | 499.91 |
| 24 | Spectral Width | 8012.8 |
| 25 | Lowest Frequency | -996.5 |
| 26 | Nucleus | 1H |

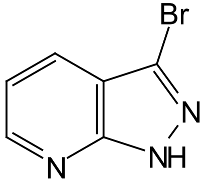


$^{1}$H NMR (500 MHz, DMSO-$d_6$) δ 8.61 (dd, $J$ = 4.6, 1.9 Hz, 1H), 8.10 (dd, $J$ = 8.1, 1.8 Hz, 1H), 7.30 (dd, $J$ = 8.2, 4.4 Hz, 1H).

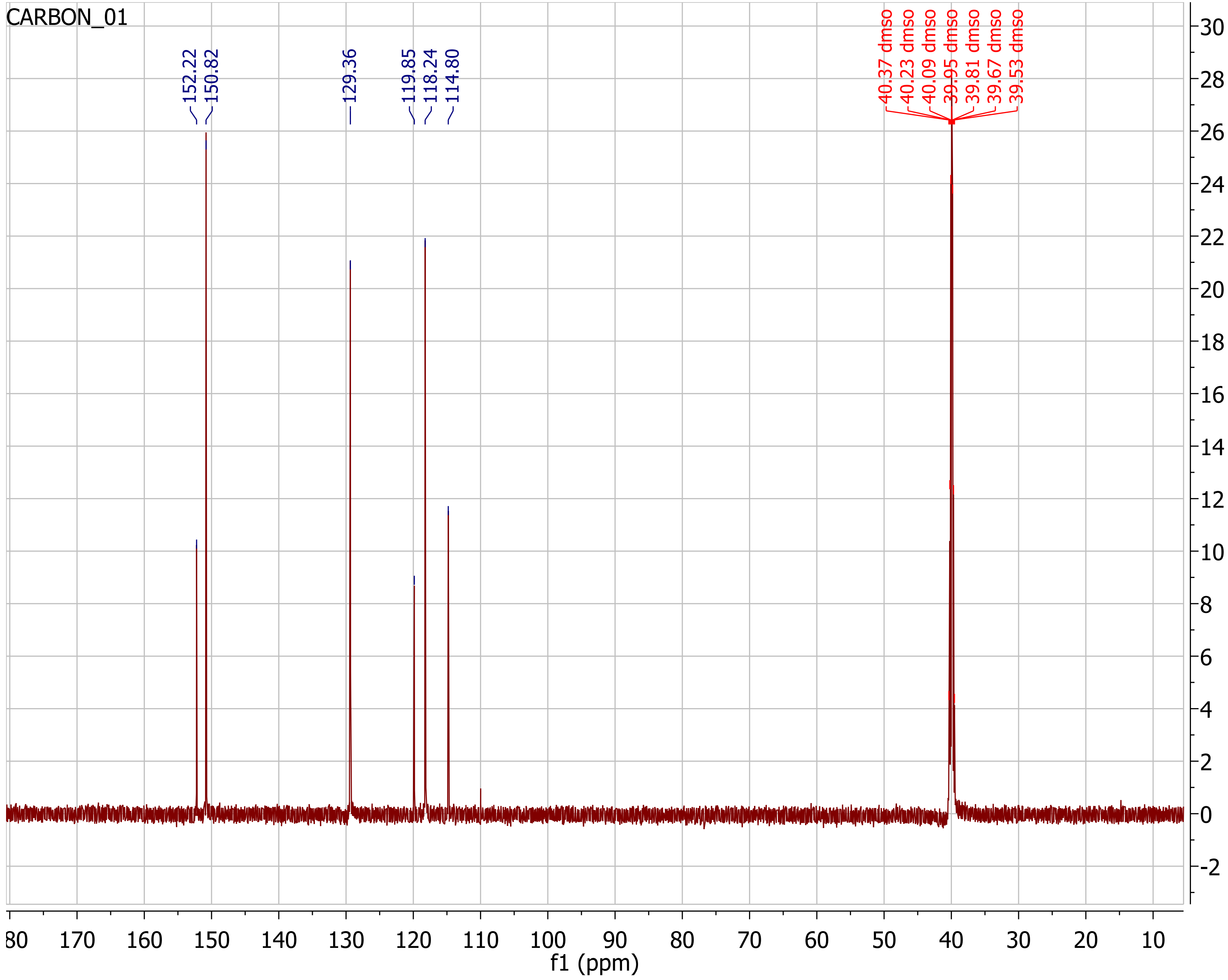


Sussex Drug Discovery Centre

| | Parameter | Value |
|---|---|---|
| 1 | Data File Name | F:/ DB Project/ NMR/ Conc C NMR/ N2118-137_01/ CARBON_01.fid/ fid |
| 2 | Title | CARBON_01 |
| 3 | Comment | |
| 4 | Origin | Varian |
| 5 | Owner | |
| 6 | Site | |
| 7 | Spectrometer | vnmrs |
| 8 | Author | |
| 9 | Solvent | dmso |
| 10 | Temperature | 25.0 |
| 11 | Pulse Sequence | s2pul |
| 12 | Experiment | 1D |
| 13 | Probe | P8891 |
| 14 | Number of Scans | 256 |
| 15 | Receiver Gain | 30 |
| 16 | Relaxation Delay | 1.0000 |
| 17 | Pulse Width | 4.1500 |
| 18 | Presaturation Frequency | |
| 19 | Acquisition Time | 0.8651 |
| 20 | Acquisition Date | 2018-01-24T11:04:47 |
| 21 | Modification Date | 2018-01-24T11:12:50 |
| 22 | Class | |
| 23 | Spectrometer Frequency | 150.81 |
| 24 | Spectral Width | 37878.8 |
| 25 | Lowest Frequency | -2417.2 |
| 26 | Nucleus | 13C |

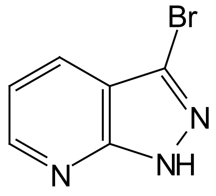

Supplement: Spectroscopic data for synthesised compounds [file rsos180333supp1.pdf]
